# Road Map for the Use of Electron Spin Resonance Spectroscopy in the Study of Functionalized Magnetic Nanoparticles

**DOI:** 10.3390/ma18122841

**Published:** 2025-06-16

**Authors:** Tomasz Kubiak, Bernadeta Dobosz

**Affiliations:** Department of Physics of Functional Materials, Faculty of Physics and Astronomy, Adam Mickiewicz University, Uniwersytetu Poznańskiego 2 Street, 61-614 Poznań, Poland; kubiakt@amu.edu.pl

**Keywords:** EPR spectroscopy, EPR imaging, magnetic nanoparticles, iron oxide, functionalization, drug delivery

## Abstract

Electron paramagnetic resonance (EPR) spectroscopy is gaining increasing recognition in research on various nanostructures. In the case of iron oxide nanoparticles, EPR measurements offer the possibility of determining the magnetic phase and the exact type (Fe_3_O_4_, γ-Fe_2_O_3_, α-Fe_2_O_3_, or a combination) of the core material. Furthermore, the EPR technique enables the study of relaxation processes, estimation of the effective and surface anisotropy constants, and assessment of the influence of sample aging on the magnetic properties of nanoparticles. The scope of the information obtained can be further expanded by utilizing spin labeling of polymer-coated nanoparticles. By analyzing the signals from the attached nitroxide, one can determine certain properties of the coating and its interactions with the environment (e.g., body fluids, cells, tissues) and also perform imaging of nanoparticles in various media. In some cases, EPR can help monitor the encapsulation of active substances and their release processes. Unfortunately, despite the enormous potential, not all of the possibilities offered by EPR are routinely used in nanoscience. Therefore, the present article aims not only to present the current applications and existing trends but also to indicate directions for future EPR research, constituting a road map.

## 1. Introduction: Basics of the EPR Technique in Nanoparticle Research

The current article is devoted to summarizing the possibilities of using electron spin resonance spectroscopy in the study of magnetic nanoparticles and their interactions with the biological environment. It discusses in detail what information can be obtained by analyzing the spectra of nanoparticle cores containing various iron oxides, e.g., magnetite or maghemite. A separate section shows advanced methods of EPR signal analysis that allow for extracting additional data from standard spectra recorded with a spectrometer operating in the X-band microwave region. The next key aspect is the presentation of various EPR studies of functionalized nanoparticles, primarily using attached spin labels such as TEMPO derivatives. Research on nanoparticles for their future biomedical applications includes their interactions with different media, such as serum and whole human blood. It is also noted that electron spin resonance spectroscopy is an ideal tool for characterizing magnetic cargo contained in various delivery systems, e.g., magnetite nanopowder constituting the payload of liquid marbles. Quantitative X-band EPR is presented, as well as the still actively developing field of imaging nanoparticles in various media using machines operating at lower microwave frequencies (L band). Since EPR spectroscopy is primarily associated with the study of free radicals, the paper also includes a description of the possibilities of determining the redox activity of nanoparticles and trapping reactive oxygen species generated in their presence in the biological environment. Attention is also drawn to the interesting application of EPR spectroscopy in cell-based research. Due to the fact that the article is intended to be a road map, the work ends with a section devoted to perspectives for future studies. It outlines the prospects for the use of electron spin resonance spectroscopy in nanoparticle research and suggests directions for planning further research.

EPR spectroscopy is a method recognized in the scientific world for the study of samples containing unpaired electrons. Description of the physical basis of this technique can be found elsewhere [1]. Nevertheless, it is worth recalling some basic information. A splitting of energy levels called the Zeeman effect occurs when the paramagnetic specimen is placed in an external magnetic field. The energy of the individual levels depends on the orientation of the spin angular momentum ***S*** and its associated magnetic dipole moment *μ_s_*, which are antiparallel to each other.

(1) $$\mu_{s}=-g\mu_{B}S$$

In the above formula, *µ_B_* denotes the Bohr magneton (*μ_B_* = 9.274009·10^−24^ [J/T]). The value of a dimensionless quantity called the *g* factor is precisely measured for a free electron: *g_e_* = 2.0023193043617 [2]. Spin is quantized with respect to the direction of the external magnetic field *B*_0_, so its projection onto the direction of this field takes only discrete values defined by the magnetic quantum number *m_s_*. Knowing the formula for the energy *E_m_* of the spin state:

(2) $$E_{m}=g\mu_{B}m_{s}B_{0}$$

one can easily determine that in the simplest case of a single electron with spin *S* = ½ (*m_s_* = ±½), there are two Zeeman energy levels:

(3) E±12=±12gμBB0

When we supply the system with quanta of electromagnetic radiation with the energy *hν*, which matches the energy difference Δ*E* between these split levels, the resonance condition is satisfied:

(4) $$\Delta E=h\nu =g\mu_{B}B$$

where *h* is a Planck constant (*h* = 6.626069·10^−34^ [J⋅s] and *ν* is the frequency of electromagnetic radiation.

Detection of this absorption is the fundamental principle of EPR spectroscopy measurements. In practice, the *g* value obtained in the experiments for various samples often differs significantly from the previously mentioned *g* value for a free electron due to the contribution of the orbital angular momentum and different local fields of atoms or molecules [3]. In metals, under the simultaneous action of both a radio-frequency electromagnetic field and a perpendicular magnetic field *B*_0_, magnetic moments of the freely diffusing conduction electrons contribute to the resultant macroscopic magnetization, and the presence of the internal interactions leads to a shift in the *g* factor value [4]. Among the number of factors that influence the results of EPR research, attention should be paid to short-range exchange interactions and long-range magnetic dipole–dipole interactions that occur in the case of nanoparticle systems [5]. Uncoated magnetic grains tend to agglomerate. For aggregated particles that are in close proximity to each other, the exchange interactions between surface atoms of adjacent particles cannot be neglected. On the other hand, in the case of surfactant-protected structures, magnetic dipole interactions are more important. The energy *E_dip_* of dipole interactions for randomly distributed ferro- or ferrimagnetic particles with the dimensions reduced to several nanometers can be expressed by the formula:

(5) $$E_{dip}\approx \frac{\mu_{0}\mu^{2}}{4\pi d^{3}},$$

where *µ* is an average magnetic moment of nanoparticles, *µ_0_* is vacuum magnetic permeability, and *d* denotes inter-particle distance [6].

The most elementary analysis of well-resolved and non-overlapping EPR spectra of magnetic nanoparticle samples includes determining parameters, such as the resonance field *B_r_*, the peak-to-peak EPR linewidth Δ*B_pp_*, the *g* factor value, and the asymmetry parameter *R*, calculated as the ratio of the positive and negative peak heights. These heights are set from the maximum or minimum to the baseline, and the value *R* = 1 indicates perfect symmetry. Certainly, a more detailed analysis of the spectra allows the calculation of more parameters and obtainment of much additional valuable information about the nanoparticle system.

## 2. EPR for Characterization of Nanoparticle Cores

For research purposes and biomedical applications, nanoparticles with core diameters ranging from a few to several dozen nanometers are most often synthesized. Iron oxides are frequently chosen for the production of nanostructures susceptible to magnetic fields. We can distinguish between magnetite (Fe_3_O_4_), maghemite (γ-Fe_2_O_3_), and hematite (α-Fe_2_O_3_) material. The first two have cubic cells and exhibit ferrimagnetic behavior, while the last shows canted antiferromagnetism and rhombohedral crystal structure [7]. Further differences result from the fact that magnetite contains both Fe^2+^ and Fe^3+^ cations, maghemite in its sublattice has Fe^3+^ cations and vacancies, while hematite includes only Fe^3+^ ions without periodic vacancies [7].

For Fe_3_O_4_ nanoparticles, a transition from ferrimagnetic to superparamagnetic phase is observed at a certain critical size, according to most sources 25 nm [8,9,10,11]. For γ-Fe_2_O_3_ nanoparticles, a value of 30 nm is mentioned [10,11]. The critical size for which this transition occurs certainly depends on the properties of the material [12]; therefore, the composition of the prepared nanoparticles should be carefully determined. Even for declared magnetite nanoparticles, one can observe the differences between samples from various sources, especially in terms of the share of the oxidized Fe^3+^ compared to Fe^2+^ atoms on the outer layer of the particles. Therefore, EPR spectroscopy should be considered a widely applicable technique for characterizing magnetic nanoparticles at different stages of their lifespan. The structure and parameters (peak-to-peak linewidth Δ*B_pp_*, signal intensity, and above all, the *g* factor) of EPR spectra can provide a lot of valuable information about samples. The g factor, called a spectroscopic spilitting factor, is characteristic of each paramagnetic center. The shape of the signal is also an important factor. For example, when the mean size of magnetite core increases, the EPR spectrum manifests itself as more asymmetric [13].

In general, in the case of ferrite nanoparticles of a few nanometers in size, one can observe a ferrimagnetic phase with well-defined remanence and coercive field at low (e.g., liquid nitrogen) temperature, a superparamagnetic phase with neither remanence nor coercive field below room temperature, and a paramagnetic phase showing a linear dependence of the magnetization on the applied magnetic field when temperature is increased above the Curie point [14]. For samples with remanent magnetization, strong dipole–dipole interactions or magnetic coupling can result in intense and broad signals attributed to ferromagnetic resonance [15]. Ferromagnetic behavior of nanoparticles is usually observed below the blocking temperature *T_B_*, whereas above this temperature, thermal fluctuations dominate over anisotropic magnetic interactions and the magnetic moments of nanoparticles exhibit superparamagnetic behavior [16]. Values of *T_B_* for magnetite nanoparticles depend on their size and synthesis method. Exemplary values are *T_B_* = 134 K for particles (ø = 11 nm) and 150 K (ø = 6 nm) [17]. Additionally, one should remember that the blocking effect varies for different measuring techniques, and in the case of EPR spectroscopy, has an influence on the recorded spectra. At blocking temperature *T_B_*, the relaxation time *τ_R_* of the magnetic moments corresponds to measuring time *τ_m_* (for X-band EPR *τ_m_* = 10^−10^ s) [16].

(6) $$T_{B}=\frac{K_{1}V}{k_{B}\ln\left(\frac{\tau_{m}}{\tau_{0}}\right)},$$

In the above equation, *k_B_* is the Boltzmann constant, *τ*_0_ represents a time constant, which is characteristic of the material (10^−9^–10^−11^ s for ferro- and ferrimagnetic nanoparticles [18]); *V* denotes the volume of the nanoparticle core, and *K*_1_ is the first-order anisotropy constant. For electron spin resonance measurements, the thermal fluctuation energy (*k_B_T_B_*) has a value close to the energy barrier *K*_1_*V* between the equilibrium positions of the nanoparticle magnetic moment [16]. Therefore, *T_B_* values obtained on the basis of resonance spectra are higher compared to those derived from longer-time-scale experiments, e.g., static magnetic susceptibility research [18].

Scientists are working to make EPR spectroscopy a fast and universal tool for recognizing the magnetic phase of tested samples. Classification can be performed based on the evaluation of the spectra and the calculated parameters (*B_r_*, *g*, Δ*B_pp_*, *R*) [19]. The ferrimagnetic spectrum is usually broad and asymmetric with a relatively low resonance field. The paramagnetic signal is definitely more narrow and rather symmetrical, with a higher resonance field, and the superparamagnetic line has intermediate parameters. Sometimes, it can be difficult to clearly indicate the appropriate ranges of parameter values for individual types of nanoparticle materials. Additionally, for some samples, a superposition of states (e.g., superparamagnetism and ferrimagnetism) can occur. Therefore, fitting of the EPR spectrum using Gaussian or Lorentzian line shapes (or their combination) can be performed to verify the possibility of co-existence of magnetic phases [19]. However, it should always be borne in mind that the EPR spectrum can be influenced by many factors, such as functionalization or interactions with the environment.

The resonance formula for magnetic nanoparticle samples includes the effective field value. This *B_eff_* is created by adding internal fields to the external spectrometer field. Depending on the size of the magnetic particles, four basic types of anisotropy are responsible for the occurrence of internal fields. Magnetocrystalline anisotropy is related to a crystallographic structure, domain structure anisotropy takes into account the orientation of the microwave field relative to the domain walls, shape anisotropy is caused by the interaction of the magnetization vector with the grain demagnetizing field, and exchange anisotropy is the result of interactions between magnetic particles [15]. The latter anisotropy may also have its source in the exchange coupling at the boundary of two phases differing in magnetic order, for example, the surface and the interior of the core of nanoparticles [20]. In the case of tiny particles, the contributions from both magnetic anisotropy and the shape-related demagnetization factor are averaged out by thermal fluctuations of the magnetic moments, resulting in the decrease in width of the superparamagnetic resonance signal [21]. When the influence of magnetocrystalline anisotropy is averaged due to motion, the line narrowing factor *κ* can be determined:

(7) $$\kappa =\frac{\tau_{sp}}{\tau_{L}}=\frac{M_{S}VB_{r}}{2\pi k_{B}T},$$

where *M_S_*—saturation magnetization, *B_r_*—resonance field, *τ_L_*—Larmor precession time, and *τ_sp_*—response time of magnetic moments to random thermal forces (*τ_sp_* << *τ_L_*) [22,23]. According to the above, it can be assumed that the EPR line width should be proportional to the volume of the uncoated particle. If the nanoparticle core diameter is reduced, the EPR signal will narrow [24]. The saturation magnetization value for bulk magnetite is *M_s_* = 480–500 emu/cm^3^ [17] and for maghemite is *M_S_* = 420 emu/cm^3^ [25], so the transformation of magnetite to maghemite in the nanoparticle core may be responsible for the decrease in average saturation magnetization. Additionally, it should be noted that for individual types of nanoparticles of different sizes, the *M_S_* values may differ from those recorded for bulk materials. Alterations in the material composition of nanoparticles, translating into their measured saturation magnetization value, may be reflected in the EPR line parameters and cause interpretation difficulties. In an article about the citrate-coated superparamagnetic iron oxide nanoparticles, the difference in X-band EPR line position (and thus the calculated *g* factor: 2.41 vs. 2.06) between tested samples was hypothetically attributed to the influence of diverse core size (9.9 vs. 5.1 nm) [26]. However, such an issue should be investigated and analyzed in more detail. Since there was a maghemite layer on the outer part of the magnetite core, it would be worth considering the share of individual materials (γ-Fe_2_O_3_ and Fe_3_O_4_) in the total volume of particles, especially when the specimens had different saturation magnetization, which was experimentally proven.

Small dimensions have a significant impact on the properties of particles, as magnetization relaxation processes are strictly dependent on the temperature and size of objects [27]. As the particle diameter decreases, the fraction of surface atoms increases relative to the fraction of core atoms. For example, for nanoparticles ø ≈ 100 nm, the surface fraction is 6% of the total number of atoms, while for nanoparticles ø ≈ 5 nm, it already reaches about 78% of all atoms [28]. Surface spins cannot be neglected when describing the structure and dynamic behavior of nanoparticles. In the case of ferrites, at temperatures below 50 K, surface spins freeze into a spin-glass-like phase and thus they have multiple configurations for any core magnetization orientation [29]. According to the numerical model, magnetization within NiFe_2_O_4_ nanoparticles with a size of 6.5 nm, is not only related to ferrimagnetically aligned core spins but also to surface spin disorder, induced by reduced coordination of the surface cations and broken exchange bonds between the surface spins [29]. It is worth mentioning that for iron oxide nanoparticles. surface anisotropy can even dominate bulk anisotropy [27,30]. Therefore, when considering the anisotropy energy for particles with a diameter of several nanometers, in addition to the contribution from the bulk material inside the core, the component *K_S_*, which is related to the discontinuity of the magnetic interaction between individual spins located on the surface, should be taken into account [23,27]. For spherical particles:

(8) $$K_{S}=\left(\frac{6}{d}\right)\;k_{s},$$

*K_s_* depends on the particle diameter *d* and the surface anisotropy constant *k_s_* [31]. Néel’s approach assumes that this kind of anisotropy is positive and uniaxial with the axis locally perpendicular to the surface. Its influence is noticeable when the particle shape deviates from the ideal sphere, but an additional contribution may also be due to surface defects or surface spin disorder [27]. Electron spin resonance experiments can be used to estimate values of effective surface anisotropy of iron oxide samples. For example, for maghemite nanoparticles with a core diameter of 10 nm, *K_s_* was 2.7·10^−2^ erg/cm^2^ at 3.5 K [27]. Further comprehensive EPR research is needed, especially in the field of magnetite nanoparticles with various core sizes. It would be appropriate to test iron oxide particles produced by the same method at a similar time and stored in identical conditions. This will exclude the influence of uneven aging of the samples. It is worth noting that in the case of Fe_3_O_4_ material, we may deal with the process of maghemitization of magnetite grains. The alteration in the Fe(II)/Fe(III) ratio in the crystal structure of the specimen can be evidenced by the change in the EPR line width and *g*-factor value, especially since the energy of magnetocrystalline anisotropy in Fe_3_O_4_ is primarily due to Fe(II) [15].

**Figure 1:**
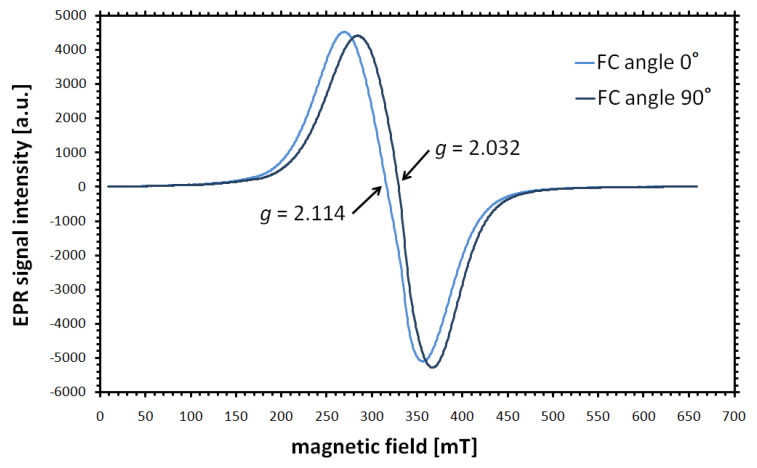
EPR spectra of PEG-coated and doxorubicin-functionalized magnetite nanoparticles (core diameter of 8–12 nm) in water. Measurements performed using Bruker EMX-10 spectrometer (Billerica, MA, USA) (sweep range 650 mT, modulation amplitude 0.5 mT, time constant 20.48 ms) after field cooling procedure (5 min in 500 mT) at 230 K for the orientations of 0° and 90° with respect to the direction of the external magnetic field (authors, unpublished).

The field cooling method, in which the sample is frozen in an external magnetic field (e.g., 0.5 or 1 T) and only then measured, is often used when determining the properties of the core of iron oxide nanoparticles. Such EPR studies, performed for nanoparticles with different functionalization and dispersed in various media, showed the temperature dependence of the resonance field *B_r_* (and thus the *g* factor), the peak-to-peak linewidth Δ*B_pp_*, and the intensity of the broad EPR line characteristic of magnetite [32,33,34]. In all these cases, the signal narrowed and its amplitude grew when the temperature was raised from 120 to 240 K. The resonance field shifted towards higher values (which means that the *g* factor decreased) as the temperature increased. For field-cooled samples, the *g* factor for the EPR signals recorded with the help of a goniometer in the orientation 0° was greater than for 90° (when the angles were measured with respect to the direction of the external magnetic field). An example of the result of such an experiment, performed for magnetite nanoparticles with a core diameter of 8–12 nm, is shown in Figure 1. The lower value of the resonance field for an angle of 0° is associated with the fact that the magnetic moments are fixed and aligned along the direction of the external magnetic field [33]. Additionally, the *B_r_* values measured for field-cooled samples with a core diameter of 10 nm were about 20 mT lower than those recorded for samples without the field cooling procedure [32]. It is worth noting that EPR measurements performed after the 0.5 T magnetic field cooling procedure showed angle-dependent *B_r_* changes not only for magnetite but also for maghemite and hematite nanoparticle samples in a glycerol matrix [7]. The approximated formula describing the angular relationship of the resonance field, taking into account the combination of both angular-dependent and -independent anisotropy fields and also the surface anisotropy field in the case of spherical ferrite nanoparticles, can be found in [35].

It is known from the literature that taking into account the reference resonance field *B_ref_* = ω/γ for ferri- and ferromagnetic particles with uniaxial anisotropy at T > 40 K, the following relations apply [36]:

(9) $$\begin{array}{l} B_{r}\left(0^{\circ }\right)=B_{ref}-B_{a}\\ B_{r}\left(90^{\circ }\right)=B_{ref}+\frac{1}{2}B_{a} \end{array},$$

Therefore, based on the values of resonance fields for the orientations 0° and 90°, it is possible to estimate the anisotropy field *B_a_* and the effective anisotropy constant *K_ef_* for magnetic nanoparticles [13,27,37]:

(10) $$B_{r}\left(90^{\circ }\right)-B_{r}\left(0^{\circ }\right)=1.5B_{a},$$

(11) $$K_{ef}=\frac{B_{a}M_{s}}{2},$$

where *M_s_*—saturation magnetization for the tested magnetic material.

It was found that both the anisotropy field and the effective anisotropy constant were larger for nanoparticles dispersed in human whole blood and serum compared to nanoparticles in water [34]. It can therefore be assumed that the properties of the medium in which the nanoparticles are present have an influence on the parameters measured by means of EPR.

The narrow EPR signal at higher temperatures results from the averaging of the effective anisotropy field, which proves the role of thermal fluctuations in the magnetic properties of the sample [24]. The resonance conditions also depend on the direction of the anisotropy axes [5]. Therefore, the EPR line not only broadens and becomes more asymmetric but also shifts towards lower field values when the sample is cooled [38]. Such thermal behavior of EPR spectra was observed many times not only for the previously mentioned magnetite nanoparticles but also for maghemite superparamagnetic structures. The examples are: 2.5 nm γ-Fe_2_O_3_ cores encapsulated in poly(propylene imine) dendrimer matrix and studied in the wide temperature range of 4.2–300 K [39], human serum albumin/γ-Fe_2_O_3_ core–shell nanoparticles (11–13 nm) tested at 80, 100, and 300 K [40] and nanoparticles containing mixed phases of rhombohedral hematite (α-Fe_2_O_3_) and cubic maghemite (γ-Fe_2_O_3_) investigated at 170–300 K [41]. Temperature changes in the spectrum parameters were proven not only in the X-band but also in the Q-band measurements of the ZnFe_2_O_4_ nanoparticles [42].

When interpreting the temperature changes of the absorption lines for magnetic particles, it is worth bearing in mind that below the blocking temperature *T_B_*, the energy of uniaxial anisotropy is larger than the energy of thermal fluctuations (*K*_1_*V* > *k_B_T*) and the absorption line is spread along the random distribution of the effective anisotropy field *B_a_* [18]. Inhomogeneous broadening of the linewidth at low temperatures results from this spread of anisotropy fields [43]. The wide signal can be treated as an envelope of the superposed EPR lines originating from the individual, variously oriented magnetic centers chaotically distributed in the sample [3]. Above *T_B_*, when *k_B_T > K*_1_*V*, uniaxial anisotropy does not significantly affect the EPR signal [18].

On the other hand, for magnetic nanoparticles, a homogeneous line broadening was also observed, the analysis of which allowed estimation of the spin–spin (transverse) relaxation time *T*_2_ [44]. When the continuous-wave X-band signal is in the form of a first derivative, one can use the equation:

(12) $$T_{2}=\frac{2\hbar }{\sqrt{3}g\mu_{B}\Delta B_{pp}},$$

It is worth pointing out that homogeneous line broadening may have many sources, including: fluctuations in the local field, dipolar coupling between particles, and spin–lattice interaction [44]. Changes in the line width with temperature result in variations in the T_2_ relaxation time, the value of which decreases as the signal broadens. For zinc ferrite nanopowder (50–70 nm), the spin–spin relaxation time determined on the basis of a broad and intense EPR signal using the abovementioned formula was of the order of 10^−12^ s [45].

When inhomogeneous line broadening occurs, the situation is more complicated, because an absorption curve can be treated as a convolution of Gaussian and Lorentzian shapes giving a Voigt profile. In such a case, the magnetic relaxation data, including spin–lattice relaxation time T_1_, might be estimated from continuous-wave measurements by means of microwave power dependence and fitted saturation curve, as shown in [46]. Recent studies on the spin–lattice coupling effect in Fe_3_O_4_ nanoparticles involved EPR spectroscopy to investigate *g*-factor differences between superparamagnetic (*g* = 2.096) and paramagnetic (*g* = 2.054) nanoparticles with identical core size of 4 nm and chemical composition [47]. Superparamagnetic particles with lattice deformations exhibited greater *g* value, and the *g*-shift was correlated with spin−lattice coupling.

A strong temperature dependence of EPR linewidth of tiny superparamagnetic iron oxide nanoparticles with homogenous size distribution may have practical importance and can be utilized in thermometry. Such an unusual application of EPR spectroscopy for monitoring of medium temperature with a spatial resolution of 5 mm, which is useful, e.g., in cryosurgery, has already been tested for nanoparticles in a solid polymer matrix in a low-temperature range from 100 to 290 K [43].

The application of electron spin resonance spectroscopy to characterize magnetic nanostructures sometimes reveals the presence of unusual EPR lines accompanying the typical broad spectrum. In a study with nanoparticles containing spinel-type iron oxide (4 nm) mineralized inside the internal cavity of Dps protein, a second signal (located around 170 mT) with smaller intensity was observed and assigned to “partially” forbidden transitions between spin states with ΔM = ±2 [38]. The same origin was attributed to a signal with *g* = 3.57 appearing in a work on iron oxide (3.5 and 8 nm) mineralized inside protein cages of bacteria *Listeria innocua* and archaeon *Pyrococcus furiosus* [48]. For surfactant-coated cobalt ferrite (CoFe_2_O_4_) samples, apart from the broad, high-intensity, and temperature dependent superparamagnetic signal (*g* = 2.1), there was a smaller line (*g* = 4.19) from isolated high-spin Fe^3+^ in the rhombic crystal field, which was attributed to α-Fe_2_O_3_ content [44]. Several EPR lines, some of which originate from forbidden transitions, were recorded for Zn_1−x_Fe_x_O nanoparticles at liquid nitrogen temperatures using X-band (9.5 GHz) and Q-band (34 GHz) spectrometers. While the Q-band measurements provided more detailed spectra and allowed more accurate determination of spin-Hamiltonian parameters, it proved to be less useful in recording forbidden transition lines, whose amplitudes decreased with rising microwave frequency [49].

Analysis of the EPR spectra of magnetite particles recorded in the temperature range 90–120 K allows the detection of the Verwey transition. During this first-order phase transition the crystallographic structure of a magnetite changes from a low-temperature monoclinic to a high-temperature cubic crystal system [50,51]. Hence, at *T* > *T_V_*, where *T_V_* is the Verwey transition temperature, magnetite has an inverse cubic spinel structure Fe(A)Fe(B)_2_O_4_. Fe^3+^ ions occupy the tetrahedral A sites, while the Fe^2+^ and Fe^3+^ ions are randomly distributed between twice as many octahedral positions (B) [52]. Below the Néel temperature (*T_N_* = 851 K), bulk magnetite exhibits ferrimagnetism, because magnetic moments of the iron ions at positions A and B are aligned antiparallel, giving a net magnetic moment corresponding to Fe^2+^ [51]. For *T* < *T_V_*, there is an order with an equal number of Fe^2+^ and Fe^3+^ ions in the B positions.

A sharp change in the line width, which was observed in some EPR studies of magnetite between 100–200 K, can be considered an indicator of the occurrence of the Verwey transition [15]. However, further research on the usefulness of EPR spectroscopy in detecting this transformation is needed, because not all studies revealed an unexpected line broadening around *T_v_*. Even though it is assumed that for magnetite *T_v_* ≈ 125 K, studies using relatively small (5–7 nm) and larger (70–80 nm) nanoparticles revealed distinct anomalies (e.g., change in the slope of the Δ*B_pp_(T)* curve) in the EPR spectra recorded between 130 K and 200 K—temperatures near and above the Verwey transition [37]. Occasionally, searching for the Verwey transition reveals other atypical spectral features. For example, deconvolution and simulation of the spectra of relatively large 50 µm magnetite grains allowed the separation of two wide (*g_ef_* = 2.4 and 4.1) and two narrow EPR signals (*g_ef_* = 2.8 and 3.4) in the temperature range of 85–125 K. This type of research should be extended to iron oxide particles with a much smaller core diameter. Interestingly, the usefulness of EPR spectroscopy in studying the Verwey transition is not limited to Fe_3_O_4_ nanoparticles. For example, paramagnetic superoxide O^2−^ entities were probed in a slow-cooling experiment with cesium sesquioxide powder [53]. In the cubic phase of Cs_4_O_6_, the EPR spectrum was broadened enormously due to rapid charge exchange compared to *τ_m_*, while in the tetragonal phase, charge localization led to an EPR signal with linewidth ΔB ≈ 0.4 T at 150 K, originating from active O^2−^ [53].

Aggregation of nanoparticles is responsible for increasing the dipole–dipole interaction between adjacent particles and consequently influences the peak-to-peak line width [26]. Therefore, the use of EPR spectroscopy for uncoated or even covering-protected samples, but stored for a longer period of time, provides an opportunity to determine the degree of their aggregation. While the presence of the dipole interactions is manifested by nanoparticle spectrum broadening, narrowing of the line may indicate the dominance of exchange interactions [54]. An example is the analysis of the shape and width of the EPR line from Mn^2+^-doped CoFe_2_O_4_ magnetic nanoparticles (Co_1−x_Mn_x_Fe_2_O_4_), which showed that an increase in manganese concentration resulted in the enhancement of superexchange interactions among Fe^3+^ cations [55]. An analogous situation occurred for magnesium- and yttrium-doped manganese ferrite nanoparticles Mg_x_Mn_1−x_Y_x_Fe_2-x_O_4_ [54] and for Mn^2+^-substituted Fe_3_O_4_ ferrofluid (Fe_1−x_Mn_x_Fe_2_O_4_) [56]. In both cases, *g*-factor and Δ*B_pp_* values decreased with increasing x. As concluded by the authors, when the dopant concentration rose, superexchange interactions were more pronounced. Also, the EPR line from paramagnetic impurities located on a solid surface narrows and takes a Lorentzian shape when a strong Heisenberg exchange dominates over the dipole–dipole interactions [24].

## 3. Advanced Methods for Analyzing X-Band EPR Spectra of Nanoparticles

As mentioned earlier, the spectra from iron oxide nanoparticles may contain various hidden components with lower amplitudes masked by the broad and intense line typical of iron oxide cores. In the simplest case, in order to visualize these smaller items, one can fit a curve (e.g., a first derivative of Lorentzian) to a broad signal from iron oxide and subtract it as a background. This procedure, performed for cubic (edge length ≈ 28 nm) crystalline iron oxide core–shell nanoparticles covered with an oleic acid layer, revealed two much narrower and smaller lines with *g* = 2.5 and *g* = 2.01 [57]. The first of them was temperature-dependent, and the position of the second remained constant as the temperature changed. Extracting the additional components from the background and then examining their behavior greatly facilitates their identification and increases knowledge about the properties of the studied system. However, sometimes more advanced processing methods are needed in order to better visualize overlapping signals.

**Figure 2:**
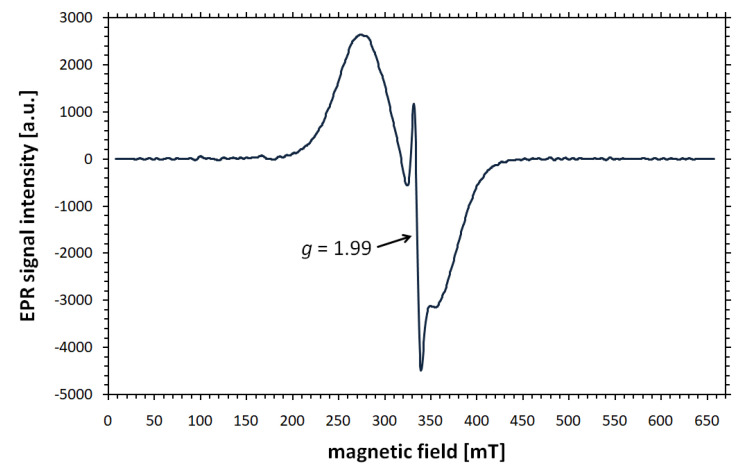
Result of the numerical processing of the X-band EPR signal of PEG-coated and doxorubicin-functionalized magnetite nanoparticles (core diameter of 8–12 nm) in water. CREM (computer resolution enhancement method) analysis allowed the extraction of a narrow line (*g* = 1.99) from the broad core signal (authors’ unpublished results).

If the shape of the EPR spectrum indicates that it has a complex structure, or a poorly outlined component appears against the background of a broad line from the nanoparticle core, the computer resolution enhancement method (CREM), can be useful. This technique, with mathematical foundations formulated by Koper and Krzyminiewski [58], facilitates the interpretation of signals consisting of many overlapping resonance lines of different widths. The mathematical formulas describing the individual stages of CREM analysis can be found in [59,60]. Although the effectiveness of the spectral resolution enhancement has been confirmed in many cases, its applications for various types of nanoparticles seem to be particularly interesting. For example, the presence of a narrow line with *g* = 1.99 and Δ*B_pp_* ≈ 7–10 mT was revealed in numerically processed X-band spectra of Endorem ferrofluid (commercially available dextran-coated Fe_3_O_4_), oleic acid-covered magnetite nanoparticles diluted in heptane [60], (3-chloropropyl)trimethoxysilane functionalized magnetite nanoparticles dispersed in toluene [61], and PEG- and silane-coated iron oxide nanoparticles in human whole blood and serum [34]. The result of the CREM analysis of the X-band EPR signal of PEG-coated and doxorubicin-functionalized magnetite nanoparticles (core diameter of 8–12 nm) in water is depicted in Figure 2. The *g* value of the narrow component is independent of the sample orientation with respect to the magnetic field and the type of surrounding environment. However, its contribution to the whole EPR spectrum depends on the size and functionalization of the nanoparticles, as well as the medium in which they are dispersed [34]. Additionally, the amplitude of the narrow signal increases significantly with rising temperature.

The phenomenon of the line with *g* = 1.99 should be thoroughly investigated, as there are many theories attempting to explain its origin. Such an unusual component, coexisting with a broad signal, was also observed in standard EPR experiments with diluted samples of magnetite [22] and maghemite nanoparticles [62]. Initially, it was attributed to the presence of very small superparamagnetic nanoparticles [22] or complexes of free Fe^3+^ in ferrofluids [63]. However, these explanations have not been confirmed by more recent studies. It is currently believed that thin line is related to phenomena occurring in the outer layer of the core. Possible sources include structural defects related to the presence of Fe^3+^ ions due to an oxidation of the surface of magnetic particles [60]. The isotropic contribution from the internal field associated with the nanoparticle surface decreases at low temperatures [28], which is consistent with the fact that the narrow line disappears when the sample is cooled. The spin fluctuations at the surface slow down, leading to the freezing of the surface spin disorder.

The occurrence of a narrow line was proven for nanoparticles with a diameter of several nanometers. However, it would be necessary to clearly establish whether there is a critical core size for which this EPR line ceases to appear. It is also worth considering the possibility of using the analysis of this signal to study the surface state of nanoparticles in various media. One should bear in mind that the use of EPR spectroscopy to study surface effects does not have to be limited to iron oxide nanoparticles. Paramagnetic defects were also detected on the outer layer of tin oxide (*g* = 1.997) [64] and zinc oxide (*g* = 2.004) nanostructures [65]. The nature of such defects, depending on the synthesis method and material characteristics, can be explored thanks to various EPR approaches, including checking microwave power saturation behavior and temperature variation of EPR signal under different atmospheric conditions. In addition, the use of higher-frequency Q-band spectroscopy can reveal additional features of spectral shapes due to better sensitivity and improved resolution [65]. It is worth mentioning that the use of EPR spectroscopy in the study of surface and core properties of nanospheres does not have to be limited to various types of superparamagnetic and ferrimagnetic particles. Scientists have also prepared nanostructures containing paramagnetic materials, e.g., Mn:CdS. Even though divalent manganese with five unpaired electrons and *S* = 5/2 is characterized by six lines of a hyperfine structure, the EPR spectrum of Mn:CdS nanoparticles was a combination of three different signals: narrow hyperfine components from Mn^2+^ ions in a CdS lattice, wider lines from manganese on the surface, and a single broad line from aggregated manganese ions in MnS clusters [66]. The EPR spectroscopic characterization of the core and surface of nanostructures may therefore also be based on dopants in the form of transition metal ions such as Fe^3+^ [67,68], Mn^2+^ [69], Zn^+^ [70], Co^2+^ [71], or Cu^2+^ [72]. In general, for in-depth studies of nanoparticles, it will be beneficial to resolve small components that are unnoticeable in conventional first-derivative EPR spectra. In addition to the previously mentioned CREM, other post-processing approaches, including convolution filtering for separation of overlapping broad and sharp spectral components [73] or an algorithm capable of splitting the superimposed signal to the basic lines [74], can be tested for nanostructures.

## 4. Applications of EPR in Nanomaterial Characterizations

### 4.1. EPR of Functionalized Nanoparticles

There is much evidence that the method of functionalizing the surface of nanoparticles influences the parameters of recorded EPR spectra. Even a simple study comparing asymmetric resonance curves recorded for iron oxide nanoparticles (g = 2.785) and their counterparts (g = 2.468) modified with poly-L-lysine cationic polymer and silk protein sericin showed that the g factor changed when a coating was used [75]. In that case, the addition of a shell resulted in a decrease in the g value. An opposite trend was observed for uncovered (*g* = 2.0396) and dextran-coated (*g* = 2.1626) magnetite nanoclusters with mean size of about 11–14 nm [76]. Interestingly, in the latter study, Δ*B_pp_* values for functionalized samples (291.2 mT) were greater than for pure nanocrystals (286.1 mT), which is not typical, since usually, adding surfactant layers causes a narrowing of the spectrum, as presented in [77,78]. It should be noted that in such cases, much depends on the distribution of nanoparticles and the type of coating material, which can influence the magnetic interactions. For example, line shape and peak-to-peak width alterations between magnetite nanoparticles with different surface modification (coating with silica, functionalized with gold or gold and poly(vinylpyrrolidone)) were attributed to differences in the chemical environment of the outer layer and the influence of the shell on magnetic properties of the entire structures [79]. Covering the nanoparticle cores with an appropriate coating causes dipolar interactions between the particles to decrease thanks to surfactant or polymer layers, which can result in reduced line width and intensity of EPR signal [77,78].

In some studies, EPR was used only for the simplest applications, such as confirming the presence of unpaired electrons in zinc oxide-coated maghemite nanoparticles [80], validating the content of iron oxide powder (50 nm) in magnetic poly(hydroxyethylmethacrylate-N-methacryloyl-(L)-histidine) nanoparticles [81] or proving the magnetic properties of Fe_3_O_4_ grains (20–50 nm) incorporated in poly(2-hydroxyethyl methacrylate–co-glycidyl methacrylate) particles with covalently immobilized L-asparaginase enzyme [82]. In the three mentioned cases, the *g*-factor values at room temperature were *g* ≈ 2.0 [80], *g* = 2.33 [81], and *g* = 2.14 [82]. The discrepancy between them, resulting primarily from different magnetic material, was most likely also influenced by distinct functionalization. This hypothesis was confirmed by a study on magnetic nanoparticles coated with carboxymethyl dextran, in which the *g* factor decreased with increasing concentrations of coating material [83]. In general, analysis of the *g*-factor value and other spectral parameters can provide useful information about nanostructures not only in terms of the core but also the coating. It is known from the literature that binding of organic molecules to the surface of ferrite nanoparticles causes the electrons involved to no longer engaging in superexchange interactions between magnetic cations [29].

An additional issue is the use of EPR spectroscopy to monitor the properties of the shell itself. EPR was introduced for the study of magnetite nanoparticles (5 nm) coated with (3-chloropropyl)trimethoxysilane and chitosan and functionalized with both a spin label 4-amino-2,2,6,6-tetramethylpiperidine-N-oxyl (4-amino TEMPO) and an antibiotic amoxicillin in an aqueous environment [32]. The form of EPR signal may reflect the degree of coating of the nanoparticle by the spin-labeled ligands. Such research was conducted, for example, using gold nanoparticles with TEMPO-substituted disulfide [84]. For low and almost uniform surface coverage by the isolated spin labels, i.e., organic molecules with an unpaired electron, a characteristic triplet occurred. However, one should remember that it was characterized by the smaller intensity of the high field line, indicating an incomplete averaging of anisotropic components of the Â and ĝ tensors due to hindered movement of the nitroxide attached to the nanoparticle surface. The increase in coating density enhanced dipole−dipole and exchange interactions among neighboring radicals. The three-line pattern merged into a broad line, which was susceptible to an exchange-narrowing effect [84]. This is yet more proof that when studying nanoparticles, attention should be paid to the influence of surface functionalization on the properties of the samples. Interactions with the surrounding environment also remain an extremely important issue.

### 4.2. EPR Study of Nanoparticles in Various Media

EPR spectroscopy can be successfully used for investigating the properties and behavior of nanoparticles present in various media and with significant surface additives. This is particularly important, because coating with a suitable polymer, e.g., chitosan or PEG, can ensure biocompatibility of nanoparticles administered to the body [85].

A very interesting issue is the application of EPR spectroscopy to study the interactions of functionalized and polymer-coated nanoparticles with biological fluids, in particular with serum, plasma, and whole human blood. Many paramagnetic centers naturally occur in these media. For example, for plasma, EPR signals from Fe^3+^ ions (*g* = 4.2) bound to transferrin and Cu^2+^ (*g* = 2.05) present in ceruloplasmin were recorded [86]. In the case of human whole blood, there were several additional lines from high-spin Fe^3+^ in methemoglobin (*g* = 5.8–6) and various low-spin ferriheme complexes (*g* = 2.21–2.91) [87]. Fortunately, EPR spectroscopy can perfectly distinguish between signals originating from ions contained in protein complexes and lines coming from iron oxide nanoparticles injected into the blood. Therefore, scientists are trying to use the EPR technique to better describe the interactions of nanoparticles with blood. Some aspects of this research were summarized in a paper devoted to the applications of EPR in medicine [88]. It has been established that the values of EPR line parameters (*g* & Δ*B_pp_*) of PEG-coated magnetite nanoparticles with a core diameter of 10 nm differ between nanoparticles in water and blood [89]. This confirms the influence of the environment on the observable properties of nanoparticles.

EPR spectroscopy can also be useful in determining the fate of nanoparticles injected into the bloodstream. Elimination kinetics of a ferrofluid containing dextran-coated Fe_3_O_4_ nanoparticles intravenously administered to rats was established by analyzing the integrated intensities of EPR lines (*g* = 2.1) from lyophilized blood and liver tissue samples collected at subsequent time points after injection [90]. If research on humans or animals is not possible for technical or bioethical reasons, a variety of hydrogels can mimic the biological environment. Comparison of the EPR spectra recorded for 8 nm nanoparticles coated with sodium oleate and polyethylene glycol 6000 and the same particles embedded in a tissue mimicking agar phantom for hyperthermia measurements revealed differences in the parameters of the broad EPR line from the core: the highest *g*-factor value and narrower spectrum were observed for pure ferrofluid [91]. This result also confirms the necessity of studying the influence of the medium on the properties of nanoparticles. The favorable characteristics of nanoparticles translate into their ability to perform the planned function after injection into the organism.

**Figure 3:**
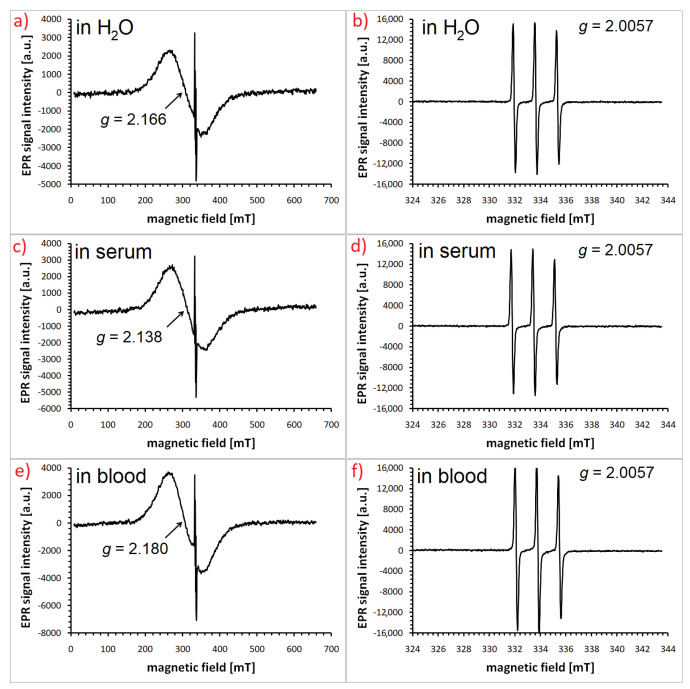
X-band EPR spectra of TEMPO-labeled and silane-coated Fe_3_O_4_ nanoparticles in: (**a**) water; (**c**) human serum; (**e**) whole human blood. The narrow-sweep-range signals from nitroxides attached to nanoparticles in: (**b**) water, (**d**) serum, and (**f**) whole blood. All spectra recorded at 293 K (authors’ unpublished results).

Functionalization of magnetic nanoparticles by attaching spin labels to their shell opens even broader perspectives for research on the interactions of these structures with the biological environment. Spin labels usually contain nitroxide moiety with unpaired electrons delocalized over the N-O^•^ group. Various derivatives of TEMPO (2,2,6,6-tetramethylpiperidine-1-oxyl) are most commonly used in nanoparticle research. Analysis of the spectrum of such labels provides a range of information not only about their microenvironment (for example, the polarity and viscosity of surrounding medium) but also about how the method of attaching organic molecules to the surface affects their mobility and interactions occurring in the outer layer of nanoparticles. As an example one can mention the work on 4-amino-TEMPO-functionalized and silane-coated Fe_3_O_4_ nanoparticles dispersed in body fluids, i.e., human serum and whole blood [92]. Examples of such EPR spectra are shown in Figure 3. In this type of study, it is important to investigate the dynamics of the rotational motion of radicals attached to nanoparticles added to the selected medium. The mobility of a spin label is reflected in the width and the shape of the nitroxide signal. In non-viscous and non-dense liquid media, we deal with fast tumbling that can be described by the rotational correlation time *τ_R_*:

(13) $$\tau_{R}=6.51\cdot 10^{-10}\Delta B_{pp0}\left(\sqrt{\frac{I_{0}}{I_{+1}}}+\sqrt{\frac{I_{0}}{I_{-1}}}-2\right),$$

In the formula shown above, which is valid for narrow hyperfine triplets characteristic of TEMPO or its derivatives, Δ*B_pp_*_0_ represents the peak-to-peak width of the central line (expressed in Gauss units: 1 Gs = 10^−4^ T) and *I_+_*_1_, *I*_0_, *I*_−1_ are the heights of the low-field, central, and high-field lines, respectively [33,93]. Other valuable information can be provided by the analysis of the dimensionless parameter *ε*. Its calculation is also based on the relative intensities of the hyperfine components:

(14) $$\varepsilon =\frac{\sqrt{\frac{I_{0}}{I_{+1}}}-1}{\sqrt{\frac{I_{0}}{I_{-1}}}-1}$$

The fact that the spin label is attached to the nanoparticle coating means that the anisotropic components of ĝ and Â tensors do not fully average out, which is manifested by the different intensity of individual triplet lines and in particular by the distortion of the high field line [93].

**Figure 4:**
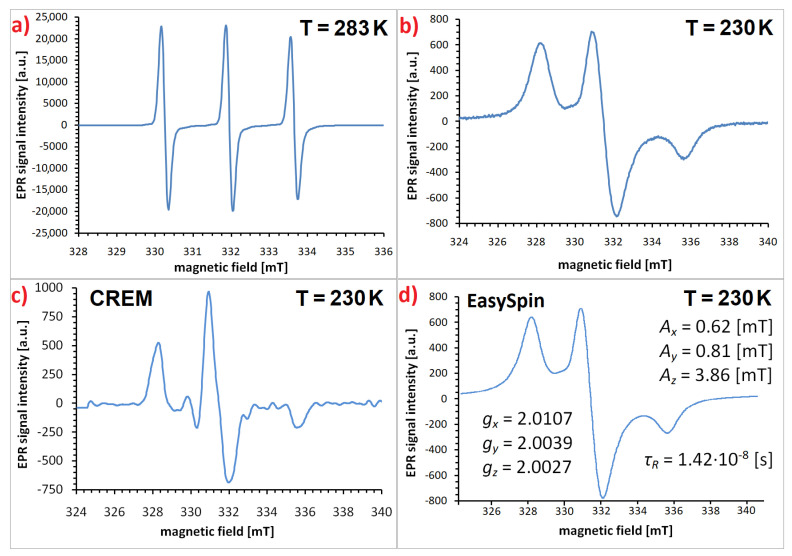
EPR spectra of the 4-amino-TEMPO spin label attached to silane-coated magnetite nanoparticles in water: (**a**) at 283 K; (**b**) at 230 K; (**c**) at 230 K after CREM analysis; (**d**) EasySpin spectrum simulation at 230 K (experimental data adapted from [34]).

In the case of highly viscous media or measurements performed at low temperatures, the spin label movement is hindered and the spectrum takes a more complex and irregular shape. In such a situation, the analysis requires EPR spectrum simulation, and the most popular program created especially for this purpose is the open-source toolbox EasySpin [94,95]. The exemplary EPR spectra of the 4-amino-TEMPO spin label attached to silane-coated magnetite nanoparticles, obtained both from experiment and simulated in EasySpin, are shown in Figure 4. From a technical point of view, it is important to remember that before simulating the spin label signal, the baseline must be corrected, as the nitroxide triplet lies on the slope of the broad signal from the nanoparticle core. Examples of the slow-motion-regime EasySpin simulations performed for TEMPO derivatives attached to magnetite nanoparticles in various media and the resulting parameters, such as tensor components *ĝ*(*g_x_, g_y_, g_z_*) and *Â* (*A_x_, A_y_, A_z_*), line width, and rotational correlation time *τ_R_*, can be found in [92,96,97].

### 4.3. EPR for Cargo Characterization

During the quality control of nanoparticle-based drug delivery systems, it is often necessary to verify whether the active substance has been properly loaded into the carrier. EPR spectroscopy can also be helpful in this matter, because even if the active substance is EPR-silent, it can be spin-labeled. As an example, one can mention a study on an anti-inflammatory drug dexamethasone labelled with 3-carboxy-2,2,5,5-tetramethyl-1-pyrrolidinyloxy (3-carboxy-PROXYL) incorporated into dendritic core–multishell nanoparticles. Room-temperature CW X-band spectra after deconvolution revealed the presence of two different signals from nitroxides. Three narrow lines came from spin-labeled drug floating freely in the solution, and the broadened component was assigned to its nanoparticle-loaded counterpart [98]. In this type of analysis, simulations performed in the EasySpin program are crucial. The rotational correlation time of the spin label depends on the motion regime and therefore reflects the microenvironment in which the sample is located. Inside the carrier, the labels attached to the drug were partially immobilized, so they were in the intermediate state between the fast tumbling and the solid-state regime.

The use of spectrometers operating in higher microwave bands to study spin-labeled payloads transported by nanoparticles remains a rare practice, probably due to the low availability of measuring equipment. Enrichment of measurements with recordings of pulsed W-band (94 GHz) spectra allowed for probing the nitroxide microenvironment with better sensitivity and precise determination of magnetic parameters—*g* and *A* matrices—that differ slightly for environments of distinct polarity [98]. Application of a W-band device for the study of nanostructured carriers also enabled determination of the localization of the TEMPO spin probe within the compartments of a lipid nanosystem [99]. The position of clearly resolved EPR lines on the magnetic field axis can reflect the character of the TEMPO microenvironment. Shifting the field down indicates rather lipophilic surroundings and shifting up a more hydrophilic neighborhood [99]. Therefore, spectrum analysis can facilitate the localization of the spin-labeled active substance and confirm whether it is enclosed in a carrier. However, it should be emphasized that studies using the most popular X-band spectrometers can also be effective in assessing payload internalization. An example is the EPR monitoring of the interaction of Gd(III) with 4-oxo-TEMPO, which allowed assessment of Gd-DTPA (diethylenetriaminepentaacetic acid) nanoparticle-loading efficiency due to the lower quenching efficiency of Gd-DTPA compared with free Gd^3+^ [100].

Even though spin labels or probes are needed for EPR-silent payloads, cargoes that contain magnetic materials can be evaluated directly. For example, X-band EPR spectroscopy enabled the characterization and differentiation of various forms of iron oxide nanoparticles, in particular: iron oxide powder, spherical magnetite-based clusters and water-based magnetic fluids and magnetosomes (magnetic nanoparticles produced by specialized bacteria), which were incorporated into liquid marbles [3].

Another situation occurs when the addition of an active substance to magnetic nanoparticles changes the EPR signal characterizing their cores. EPR studies showed that the *g* value measured for iron oxide nanoparticles (8–12 nm) coated with poly(ethylene glycol)-block-poly(4-vinylbenzylphosphonate) copolymer and loaded with doxorubicin was higher than for analogous particles without a chemotherapeutic agent attached to their surface. The increased *g* factor indicates a greater anisotropic field, which can be caused by minor changes in the subsurface region of the core [101].

### 4.4. EPR in the Study of Redox Activity of Nanoparticles

EPR spectroscopy is a unique method for direct detection and identification of free radicals in various samples, also in biological systems. Even short-lived intermediates can be captured after rapid freezing of the specimen by low-temperature EPR or more simply using a spin trapping technique [102]. Spin traps are diamagnetic compounds that are able to capture short-lived radicals and thus form stable paramagnetic spin adducts easily detectable using EPR spectroscopy. Interestingly, qualitative and quantitative analysis of reactive oxygen species (ROS) is also useful in research on nanomaterials, especially in determining their possible toxicity to biomolecules such as nucleic acids, lipids, or proteins [103]. One the one hand, the effect of free radicals may be harmful to healthy tissues, but on the other hand, it can be desirable in the case of cancer cells. It is therefore worth monitoring it with EPR.

EPR spectroscopy combined with the spin trapping technique has yielded significant results in the study of radical reactions induced in the presence of nanoparticles. Analysis of the EPR spectra of DMPO-^•^OH (hydroxyl radicals trapped by 5,5-dimethyl-1-pyrroline-N-oxide) proved that an immunogenic cancer cell death nanoinducer made of iron oxide nanoparticles and curcumin loaded into dendritic mesoporous organosilica nanostructures was able to trigger the Fenton reaction and decompose H_2_O_2_, resulting in the generation of hydroxyl radicals (^•^OH) [104]. The same spin trap revealed that the modification of magnetite nanoparticles with graphene oxide reduced the amount of ^•^OH originating from an Fe_3_O_4_-mediated Fenton reaction, and thus minimized mesenchymal stem cell damage caused by reactive oxygen species [105]. DMPO captured hydroxyl radicals converted from H_2_O_2_ in the presence of PEG-coated γ-Fe_2_O_3_ nanoparticles embedded in the bilayer of liposomes [106]. In the case of dual-modal contrast agent in the form of 4-amino-TEMPO-labeled Fe_3_O_4_ nanoparticles, EPR spectroscopy revealed not only confirmation of the attachment of the nitroxide radical but also, when using a DMPO spin trap, revealed the generation of ^•^OH as a result of magnetic hyperthermia treatment [107]. Investigations of oxidation processes with hydrogen peroxide as an intermediate also included DMPO-assisted trapping of hydroxyl radicals that were released during nuclease activity (toward the plasmid DNA) of copper-decorated and gold-coated Fe_3_O_4_ nanoparticles [108].

Thanks to the EPR method it was possible to understand the role played by various divalent ions in the peroxidase-like catalytic activity of ferrite nanoparticles [109]. Such an enzyme-like action of γ-Fe_2_O_3_ and Fe_3_O_4_ internalized into cells containing H_2_O_2_ was also tested at pH 4.8 and 7.4, which reflected the conditions in lysosomes and cytosol, respectively [110]. Although EPR spectroscopy seems to be an ideal tool for this type of study, it should be remembered that relatively weak DMPO-•OH signal is superimposed by the broad and intense line from the core of superparamagnetic particles. Therefore, the spectral subtraction procedure must be carried out to correctly identify the reactive oxygen species such as hydroxyl (•OH) or perhydroxyl (•OOH) radicals.

In addition to iron-containing nanostructures, other metal oxide nanoparticles (e.g., cerium oxide) or structures grafted with low-molecular-weight antioxidants also exhibit redox activity. The broadening of the EPR line and the loss of the hyperfine structure of stable and water-soluble spin-probe dialkyl nitroxide can prove the catalase activity of nanomaterials by following O_2_ formation [111]. Singlet-oxygen generation in dopamine methacrylamide containing microgels during Fe_3_O_4_ nanoparticle-induced catechol oxidation was also detected by EPR for a wide pH range [112]. The examples can be multiplied, which proves the huge potential of the EPR technique.

It is also worth adding that tracking of oxidative stress and total redox capacity of cells and tissues can be facilitated by quantum sensors, as demonstrated for quantum dots coated with cyclodextrin functionalized with two kinds of nitroxide (paramagnetic TEMPO or diamagnetic TEMPOH) [113].

### 4.5. EPR Imaging of Nanoparticles

Electron paramagnetic resonance imaging (EPRI) as a technique for determining the spatial distribution of unpaired electrons in a sample is still under intensive development. A detailed introduction to the EPRI method, its operation, and image acquisition principles can be found elsewhere [114]. While traditional EPR spectroscopy gives a signal averaged over specimen volume, application of field gradients in three dimensions enables the spatial encoding of the signal position. It should be mentioned that due to the fact that the electron spin after excitation returns to equilibrium much faster (ns to μs) than the nuclear spin (ms to s), EPR imaging requires the use of larger gradients than NMR [115].

From a series of spectra recorded with a precisely ascertained location, two- or three-dimensional images can be reconstructed. It is worth considering the usefulness of EPRI in various types of research on the biomedical applications of nanoparticles. Due to the fact that the direct use of the broad EPR signal from the magnetite core for image reconstruction may be difficult, it is worth taking advantage of the possibilities offered by the spin-labeling technique.

**Figure 5:**
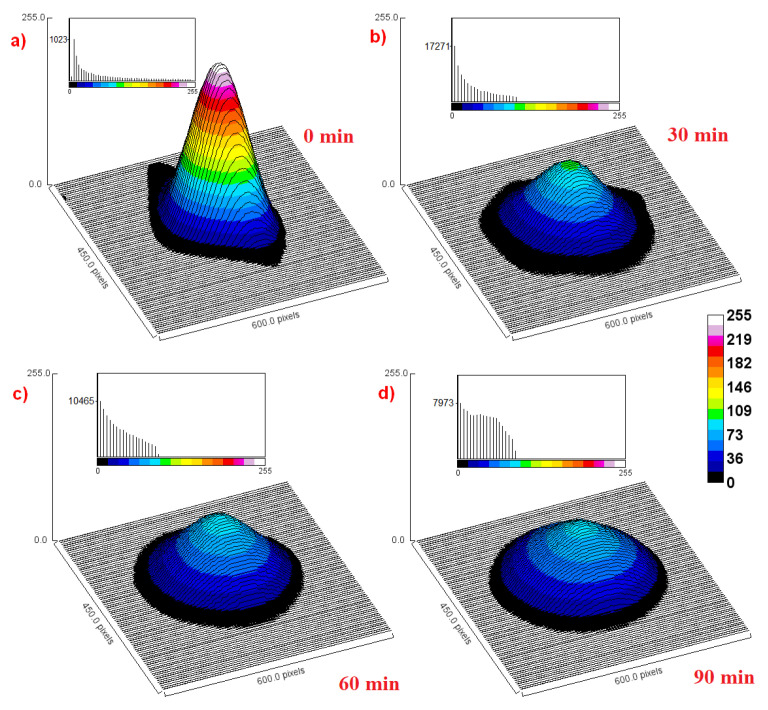
Results of EPR imaging of PEG-coated and 4-amino-TEMPO-functionalized Fe_3_O_4_ nanoparticles (core diameter 14 nm) in hydrogel (sodium alginate and calcium chloride). Measurements were carried out using an L-band (1 GHz) continuous-wave Bruker ELEXSYS E540L spectrometer (field gradient 1 mT/cm) in 30 min time intervals. Pseudocolor surface plots show the changes in the signal intensity distribution of 10 mL of the nanoparticle dispersion (5 mmol/mL) in the sample over time: (**a**) 0 min, (**b**) 30 min, (**c**) 60 min and (**d**) 90 min (authors’ unpublished results).

When spin labels are permanently attached to the surface of nanoparticles, it is feasible to use L-band EPR imaging to investigated the collective behavior of such structures in various media, including a biological environment. L-band spectrometers operate at a lower microwave frequency of 1–1.2 GHz, which is safe for biomedical applications and less affected by the water content [116]. This even makes it possible to conduct EPR research on small animals in vivo. However, it is definitely easier to utilize a tissue-mimicking phantom. EPR imaging (EPRI) based on 1 GHz continuous-wave spectrometry with a magnetic field gradient of 1 mT/cm has already been used to track the diffusion of TEMPO-labeled PEG-coated Fe_3_O_4_ nanoparticles injected into standardized (sodium alginate and calcium chloride) hydrogel [33]. The results of a similar L-band experiment are graphically presented in Figure 5. EPR imaging is therefore a useful tool for determining the spatial distribution of nanoparticles in porous media, and the measurement results facilitate the calculation of parameters such as diffusion flux, concentration gradient, and diffusion coefficient. In the future, it seems reasonable to use L-band EPR imaging to study the migration and changes in the distribution of spin-labeled and biocompatible polymer-coated nanoparticles after their injection into tissues. The next step will be to perform such measurements in vivo using tumor-bearing mice.

Interestingly, the determination of nanoparticle distribution in a sample can be performed even without the use of an advanced imaging system and gradient coils. Under certain conditions, an X-band spectrometer and a system allowing measurements of samples at different linear positions inside the cavity can be used as a substitute. Such a measurement system was employed in a study evaluating the diffusion of functionalized magnetite nanoparticles forced by an inhomogeneous magnetic field [117]. The frozen samples were moved in 0.5 mm steps under the control of a micrometer screw, and the EPR spectrum was recorded for each position. After calculating the integral intensities of the spectra and performing computational analyses, it was possible to visualize the nanoparticle concentration in individual sample layers, i.e., to determine the linear change in particle concentration in the specimen and visualize it graphically [117].

### 4.6. Quantitative EPR of Nanoparticles

In the context of studying the distribution of nanoparticles and attached spin labels in a sample, it is worth mentioning another possibility offered by electron spin resonance spectroscopy. So-called quantitative EPR can be used for the assessment of the number of unpaired electrons and thus the content of magnetic centers in the sample. For this purpose, after subtracting any possible background, the signal recorded in the form of the first derivative is integrated twice in order to obtain the area under the absorption curve. The integrated intensities of the signals from a tested sample and a standard containing a known number of spins are compared, which allows for a quantitative assessment [118]. In the field of medical nanotechnology, this creates the opportunity to check the number of magnetic nanoparticles in cells, tissues, or medicinal products. It is worth mentioning that in some work, e.g., [119], only an approximate estimation of the number of unpaired electrons in the sample was performed by multiplying the signal intensity and the square of the peak-to-peak line width. Although such simplification can significantly accelerate the analysis, accurate continuous-wave quantitative EPR should be based on a proper double-integration method following all the procedures included in [120].

### 4.7. Cell-Based EPR Research

In recent years, research on the interaction of magnetic nanoparticles with various cells has become increasingly popular. The mechanisms and the efficiency of internalization of nanostructures into cells, e.g., by endocytosis, are particularly frequently studied, because it is crucial to verify whether submicrometer-sized drug carriers reach intracellular targets. The use of EPR spectroscopy supported by spin labeling can significantly facilitate such investigations. It is therefore worth mentioning a few examples of EPR experiments with cell cultures, tissues, and laboratory animals. X-band EPR measurements allowed the determination of the concentration of Fe_3_O_4_ nanoparticles (3–9 nm) encapsulated in liposomes and administered to Lewis carcinoma-bearing mice. These quantitative biodistribution studies of magnetoliposomes were based on double integration of the broad EPR signal (*g* = 2.32) recorded at 77 K for lung, liver, and tumor tissue samples taken from decapitated animals [121]. A similar study, conducted on starch-coated iron oxide nanoparticles injected into glioma-bearing rats, showed that EPR spectroscopy of cryogenic samples (145 K) is more robust than coupled plasma optical emission spectroscopy and provides greater sensitivity in biodistribution analyses [122]. Also a presence of dimercaptosuccinic acid-coated magnetite nanoparticles administered to rats was proved in various lyophilized samples of internal organs of tested animals by dint of EPR measurements [123]. Among the examples concerning research on animals, it is also worth mentioning ex vivo EPR quantification of biodistribution of PEG–PLA-coated cobalt ferrite composite particles in vital organs (lungs, heart, kidneys, liver, and spleen), joint tissues, and lymph nodes of healthy and osteoarthritic rats [124]. Studies using redox polymeric nanoparticles with nitroxide radicals protected by compartmentation demonstrated that these structures were not internalized into healthy blood cells of rats and additionally exhibited reactive oxygen species-scavenging activity outside the cells [125]. In the context of studies on biological samples, it is worth emphasizing the serious advantage of EPR spectroscopy. In comparison with other popular analytical methods such as inductively coupled plasma optical emission spectrometry, which are not able to discriminate endogenous iron from exogenous iron oxide nanoparticles in tissues, EPR spectroscopy can easily distinguish between different chemical forms of iron [115].

**Figure 6:**
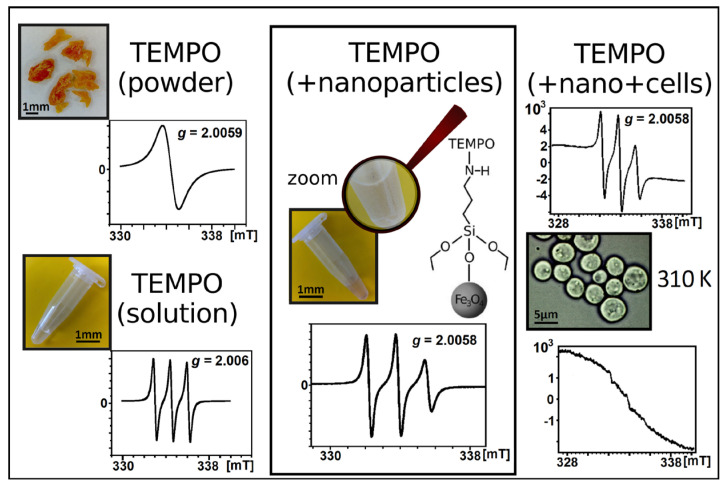
EPR spectroscopy supported by the spin-labeling technique can facilitate the study of the interaction of nanoparticles with biological surroundings. The signal from nitroxide (TEMPO or its derivatives) clearly varies depending on the environment. The powder spectrum has the form of a single line. In solution, we observe a characteristic triplet. When TEMPO is attached to the nanoparticle, the high field line is distorted due to hindered rotation of the nitroxide and the anisotropic components of the ĝ and Â tensors not being fully averaged. EPR is an efficient tool for monitoring nanoparticle endocytosis and redox processes occurring inside cells. For example, incubation of yeast in the presence of TEMPO-functionalized Fe_3_O_4_ particles performed at 310 K results in the recombination of spin labels (figure based on authors’ own research).

It should not be forgotten that the technique of spin labeling of nanoparticles may also be extremely useful in cellular research. Figure 6 illustrates this type of application. Tracking the fate of nitroxides during experiments can reveal whether the drug delivery system reaches its destination and fulfills predefined tasks at the target site. For example, nitroxide spin labels, which are often attached to nanoparticles, have the ability to cross the epidermal barrier and penetrate human skin [126]. However, both at the surface and inside the skin, due to the activity of the antioxidant system, TEMPO degrades rapidly by being reduced to EPR-silent hydroxylamine [99]. This opens up the possibility of testing the leakproof capacity of coatings when the label is encapsulated (it does not degrade in the skin environment) or confirming the controlled release of the labeled substance (gradual disappearance of the signal from nitroxide). The penetration of both nanocrystals and base-cream formulation of corticosteroid dexamethasone covalently linked with a paramagnetic probe of 3-(carboxy)-2,2,5,5-tetramethyl-1-pyrrolidinyloxy into porcine skin was tracked ex vivo using EPR spectroscopy. A number of differences in the shape of the EPR signal were observed, dependent not only on the type of formulation but also on the incubation time and location (water or dermis of healthy and barrier-disrupted skin) [127]. It would be worthwhile to conduct this type of research using spin-labeled magnetic nanoparticles with various polymer shells.

Relatively often, EPR studies of nanoparticle interactions with biological matter are performed on yeast cells. There are many reasons for this, including yeast cultures being stable, nonpathogenic, widespread in nature, capable of dividing every few hours, and easy to grow, maintain, separate, and scale up [128]. Moreover, these unicellular fungi hold the status of an eukaryotic model organism in molecular biology. Their advantages have been used in many scientific projects so far, so it is worth recalling a few of them.

The interactions between yeast cells and silica- and dextran-coated Fe_3_O_4_ nanoparticles functionalized with spin labels (various derivatives of TEMPO) were investigated in terms of endocytosis and redox processes occurring inside this cells. It was found that the length of the incubation process (performed at 310 K and resulting in recombination of spin labels) had an impact on the structure and parameters of the EPR spectra recorded at 240 K when molecular dynamics were slowed [129]. Additional studies in this area conducted on breast cancer cells and human microvascular endothelial cells proved that the EPR signal from the spin label attached to nanoparticles varied depending on the location: outside the cell, on the cell membrane, or inside the cellular structures. However, it should be remembered that the differences are only visible for frozen samples, because at room temperature molecular dynamics are very high and the impact of interactions is averaged [97]. EPR can also be an efficient tool for monitoring external magnetic field-assisted nanoparticle internalization into cells, as shown for bakery yeast cells incubated with TEMPOL-functionalized magnetite nanoparticles under the influence of rotating neodymium magnets [130]. Finally, it is worth mentioning an example of an unusual application of electron paramagnetic resonance for heating, demonstrated by scientists who assessed the survival fraction of yeast cells in a hyperthermia experiment with superparamagnetic magnetite nanoparticles [131]. Taking into account the previously enumerated advantages of yeast, further research with these microorganisms and magnetic nanoparticles can be expected.

## 5. Perspectives for Future Studies

Although there are many results of EPR studies of magnetic nanoparticles in the literature, even by combining data obtained from numerous works, it is difficult to create a universal standard that would allow assignment of the shape of the spectra and specific values of the EPR line parameters to a given type of nanoparticle core in a specific environment. This is because individual studies are performed for nanoparticles with assorted core dimensions and various surface functionalization, dispersed in different media and measured in varying temperature ranges. Furthermore, even if scientists declare a specific material, such as magnetite or maghemite, nanoparticles are often produced using non-standard methods and stored under different conditions until the measurement is performed, sometimes for extended periods of time. Therefore, they are susceptible to changes in the outer-core layer to varying degrees. All of these factors affect the results of EPR measurements. The authors of the present article know from their own experience that even purchasing Fe_3_O_4_ nanoparticles from a reputable manufacturer does not guarantee that the product will have the declared magnetic characteristics. Hence, extensive research is needed to systematize the knowledge about EPR spectra of magnetic nanoparticle cores. Such studies must be carried out in the widest possible temperature range and use nanoparticles of a specific type (e.g., Fe_3_O_4_ or γ-Fe_2_O_3_) with many core diameter values (each with the smallest possible deviation) produced according to a specific procedure and stored in a particular medium for a similar period of time. Such experimental rules certainly pose a technical and organizational challenge, but they will allow for the creation of a representative database of EPR spectrum parameters. Comparison with these standard values will enable the routine use of EPR spectroscopy to characterize nanoparticles in terms of their magnetic phase, content of individual types of iron oxide materials, occurrence of surface effects, and aging processes. It should be expected that advanced signal processing and analysis methods, such as CREM, will be an added value in this type of investigation. In the near future, the role of spectrometers operating in microwave ranges other than the X band will gradually increase. Attaching spin labels to nanostructures will not only allow for their imaging by means of L-band machines but also for determining the properties of the shell and its interactions with the nearest environment. We can expect a continuation of scientific work on the interaction of functionalized nanoparticles with body fluids and various tissues. For example, further research is required to determine the exact site and mechanisms of recombination of spin labels attached to nanoparticles during their transport into cells. EPR studies may help to find whether this process occurs in the cytosol or inside specific cellular organelles, such as mitochondria.

The possibilities offered by electron spin resonance spectroscopy in the study of magnetic nanoparticles seem to be very broad, and one can only hope that scientists will not lack inspiration and will have increasingly better technical solutions in the field of EPR spectroscopic tools.

## 6. Conclusions

Potential applications of magnetic nanoparticles, such as drug carriers, MRI contrast agents, or hyperthermia inducers, require the proper verification of these small structures, in terms of both their magnetic material content and their interaction with the biological environment. Among the physical methods of characterization of nanostructures, EPR spectroscopy deserves special attention. As shown by numerous studies, this technique can provide interesting information about various types of magnetic nanoparticles, primarily those containing iron oxides in their core. Thanks to electron spin resonance, it is possible to distinguish whether the core contains magnetite, maghemite, hematite, or a mixture of iron oxides, as well as to determine the magnetic phase of the material used. Additionally, the analysis of signals from nanoparticle cores provides information on their size, processes occurring on the surface, the contribution of different types of anisotropy, and the influence of sample aging on the change in magnetic properties. The scope of information obtained from complex resonance lines can be further expanded by the use of advanced signal processing methods such as CREM. In the case of functionalized nanoparticles, the use of attached spin labels such as TEMPO and its derivatives is also extremely useful. The analysis of signals from nitroxides allows the study of interactions of the nanoparticle coating with the microenvironment and imaging particles in various biological media using spectrometers operating in lower microwave bands. EPR measurements of nanoparticle dispersions are typically performed over a wide temperature range in various media, such as water, body fluids, tissues, and cell colonies. Despite its enormous potential, the capabilities of EPR spectroscopy are not yet fully exploited in the study of nanostructures. The reasons include limited availability of spectrometers, a relatively small number of specialists skilled in interpreting complex spectra, and above all, still incomplete knowledge of the subject. Therefore, further research is needed, the direction of which is indicated in the dedicated section of this road map.

## Data Availability

No new data were created or analyzed in this study.

## Abbreviations

The following abbreviations are used in this manuscript:

CREM	computer resolution enhancement method
DMPO	5,5-dimethyl-1-pyrroline-N-oxide
EPR	electron paramagnetic resonance
EPRI	electron paramagnetic resonance imaging
ROS	reactive oxygen species
TEMPO	2,2,6,6-tetramethylpiperidine-1-oxyl

## References

[1] Junk M.J.N., Junk M.J.N. (2012). Electron Paramagnetic Resonance Theory. Assessing the Functional Structure of Molecular Transporters by EPR Spectroscopy.

[2] Odom B., Hanneke D., D’Urso B., Gabrielse G. (2006). New Measurement of the Electron Magnetic Moment Using a One-Electron Quantum Cyclotron. Phys. Rev. Lett..

[3] Bielas R., Kubiak T., Molcan M., Dobosz B., Rajnak M., Józefczak A. (2024). Biocompatible Hydrogel-Based Liquid Marbles with Magnetosomes. Materials.

[4] Díaz-Pardo R., Valenzuela R., Osman B.S. (2015). Characterization of Magnetic Phases in Nanostructured Ferrites by Electron Spin Resonance. Advanced Electromagnetic Waves.

[5] Shukla A.K. (2018). EMR/ESR/EPR Spectroscopy for Characterization of Nanomaterials.

[6] Mørup S., Hansen M.F., Frandsen C. (2010). Magnetic interactions between nanoparticles. Beilstein J. Nanotechnol..

[7] Can M.M., Coşkun M., Fırat T. (2012). A comparative study of nanosized iron oxide particles; magnetite (Fe_3_O_4_), maghemite (γ-Fe_2_O_3_) and hematite (α-Fe_2_O_3_), using ferromagnetic resonance. J. Alloys Compd..

[8] Nguyen M.D., Tran H.-V., Xu S., Lee T.R. (2021). Fe_3_O_4_ Nanoparticles: Structures, Synthesis, Magnetic Properties, Surface Functionalization, and Emerging Applications. Appl. Sci..

[9] Liang J., Ma H., Luo W., Wang S. (2013). Synthesis of magnetite submicrospheres with tunable size and superparamagnetism by a facile polyol process. Mater. Chem. Phys..

[10] Busquets M.A., Fernández-Pradas J.M., Serra P., Estelrich J. (2020). Superparamagnetic Nanoparticles with Efficient Near-Infrared Photothermal Effect at the Second Biological Window. Molecules.

[11] Ferreira M., Sousa J., Pais A., Vitorino C. (2020). The Role of Magnetic Nanoparticles in Cancer Nanotheranostics. Materials.

[12] Kolhatkar A.G., Jamison A.C., Litvinov D., Willson R.C., Lee T.R. (2013). Tuning the Magnetic Properties of Nanoparticles. Int. J. Mol. Sci..

[13] Vargas J.M., Lima E., Zysler R.D., Duque J.G.S., De Biasi E., Knobel M. (2008). Effective anisotropy field variation of magnetite nanoparticles with size reduction. Eur. Phys. J. B.

[14] Flores-Arias Y., Vázquez-Victorio G., Ortega-Zempoalteca R., Acevedo-Salas U., Ammar S., Valenzuela R. (2015). Magnetic phase transitions in ferrite nanoparticles characterized by electron spin resonance. J. Appl. Phys..

[15] Fischer H., Luster J., Gehring A.U. (2007). EPR evidence for maghemitization of magnetite in a tropical soil. Geophys. J. Int..

[16] Berger R., Bissey J.-C., Kliava J., Daubric H., Estournès C. (2001). Temperature dependence of superparamagnetic resonance of iron oxide nanoparticles. J. Magn. Magn. Mater..

[17] Ozkaya T., Toprak M.S., Baykal A., Kavas H., Köseoğlu Y., Aktaş B. (2009). Synthesis of Fe_3_O_4_ nanoparticles at 100 °C and its magnetic characterization. J. Alloys Compd..

[18] Ennas G., Musinu A., Piccaluga G., Zedda D., Gatteschi D., Sangregorio C., Stanger J.L., Concas G., Spano G. (1998). Characterization of Iron Oxide Nanoparticles in an Fe_2_O_3_−SiO_2_ Composite Prepared by a Sol−Gel Method. Chem. Mater..

[19] Subin J.P., Jacob M.M. (2021). Determination of ferrimagnetic and superparamagnetic components of magnetization and the effect of particle size on structural, magnetic and hyperfine properties of Mg_0.5_Zn_0.5_Fe_2_O_4_ nanoparticles. J. Alloys Compd..

[20] Berkowitz A.E., Takano K. (1999). Exchange anisotropy—A review. J. Magn. Magn. Mater..

[21] Kliava J., Berger R. (1999). Size and shape distribution of magnetic nanoparticles in disordered systems: Computer simulations of superparamagnetic resonance spectra. J. Magn. Magn. Mater..

[22] Sharma V.K., Waldner F. (1977). Superparamagnetic and ferrimagnetic resonance of ultrafine Fe_3_O_4_ particles in ferrofluids. J. Appl. Phys..

[23] Hsu K.H., Wu J.H., Huang Y.Y., Wang L.Y., Lee H.Y., Lin J.G. (2005). Critical size effects on the magnetic resonance in Fe_3_O_4_ nanoparticles. J. Appl. Phys..

[24] Atsarkin V.A., Noginova N. (2020). Electron Spin Resonance on the Border Between Para- and Ferromagnetism: Quantum versus Classical. Appl. Magn. Reson..

[25] Figueiredo L.C., Lacava B.M., Skeff Neto K., Pelegrini F., Morais P.C. (2008). Magnetic resonance study of maghemite-based magnetic fluid. J. Magn. Magn. Mater..

[26] Atrei A., Mahdizadeh F.F., Baratto M.C., Scala A. (2021). Effect of Citrate on the Size and the Magnetic Properties of Primary Fe_3_O_4_ Nanoparticles and Their Aggregates. Appl. Sci..

[27] Gazeau F., Bacri J.C., Gendron F., Perzynski R., Raikher Y.L., Stepanov V.I., Dubois E. (1998). Magnetic resonance of ferrite nanoparticles:: Evidence of surface effects. J. Magn. Magn. Mater..

[28] Vázquez-Victorio G., Acevedo-Salas U., Valenzuela R., Orhan Y. (2013). Microwave Absorption in Nanostructured Spinel Ferrites. Ferromagnetic Resonance.

[29] Kodama R.H., Berkowitz A.E., McNiff J.E.J., Foner S. (1996). Surface Spin Disorder in NiFe_2_O_4_ Nanoparticles. Phys. Rev. Lett..

[30] Perzynski R., Raikher Y.L., Fiorani D. (2005). Effect of Surface Anisotropy on the Magnetic Resonance Properties of Nanosize Ferroparticles. Surface Effects in Magnetic Nanoparticles.

[31] Bødker F., Mørup S., Linderoth S. (1994). Surface effects in metallic iron nanoparticles. Phys. Rev. Lett..

[32] Dobosz B., Krzyminiewski R., Schroeder G., Kurczewska J. (2014). Electron paramagnetic resonance as an effective method for a characterization of functionalized iron oxide. J. Phys. Chem. Solids.

[33] Krzyminiewski R., Kubiak T., Dobosz B., Schroeder G., Kurczewska J. (2014). EPR spectroscopy and imaging of TEMPO-labeled magnetite nanoparticles. Curr. Appl. Phys..

[34] Kubiak T. (2016). EPR Study of Functionalized Magnetite Nanoparticles in Serum and Whole Human Blood (Badanie Metodą EPR Funkcjonalizowanych Nanocząstek Magnetytu w Surowicy i Pełnej Krwi Ludzkiej). Ph.D. Thesis.

[35] Bakuzis A.F., Morais P.C., Pelegrini F. (1999). Surface and exchange anisotropy fields in MnFe_2_O_4_ nanoparticles: Size and temperature effects. J. Appl. Phys..

[36] Upadhyay R.V., Parekh K., Mehta R.V. (2003). Spin-glass transition in a model magnetic fluid: Electron spin resonance investigation of Mn_0.5_Zn_0.5_Fe_2_O_4_ nanoparticles dispersed in kerosene. Phys. Rev. B.

[37] Nikiforov V.N., Koksharov Y.A., Polyakov S.N., Malakho A.P., Volkov A.V., Moskvina M.A., Khomutov G.B., Irkhin V.Y. (2013). Magnetism and Verwey transition in magnetite nanoparticles in thin polymer film. J. Alloys Compd..

[38] Cini A., Ceci P., Falvo E., Gatteschi D., Fittipaldi M. (2017). An EPR Study of Small Magnetic Nanoparticles. Z. Phys. Chem..

[39] Domracheva N.E., Vorobeva V.E., Gruzdev M.S., Shvachko Y.N., Starichenko D.V. (2017). EPR detection of presumable quantum behavior of iron oxide nanoparticles in dendrimeric nanocomposite. Inorg. Chim. Acta.

[40] Ganguly S., Grinberg I., Margel S. (2021). Layer by layer controlled synthesis at room temperature of tri-modal (MRI, fluorescence and CT) core/shell superparamagnetic IO/human serum albumin nanoparticles for diagnostic applications. Polym. Adv. Technol..

[41] Singh S., Goswami N. (2021). Structural, magnetic and dielectric study of Fe_2_O_3_ nanoparticles obtained through exploding wire technique. Curr. Appl. Phys..

[42] Raita O., Popa A., Toloman D., Badilita V., Piticesu R., Giurgiu L.i. (2015). Superparamagnetic behavior of ZnFe_2_O_4_ nanoparticles as evidenced by EPR. J. Optoelectron. Adv. Mater..

[43] Lin P.-Y., Chalise D., Cahill D.G. (2024). Improving nuclear magnetic resonance and electron spin resonance thermometry with size reduction of superparamagnetic iron oxide nanoparticles. Phys. Rev. Appl..

[44] Chandekar K.V., Kant K.M. (2018). Estimation of the spin-spin relaxation time of surfactant coated CoFe2O4 nanoparticles by electron paramagnetic resonance spectroscopy. Phys. E Low-Dimens. Syst. Nanostruct..

[45] Ashok K., Usha P., Nagaraju R., Ramesh T., Kumar N.P., Sulaiman G.M. (2024). Multifunctional Characterization and Anticancer Properties of Magnetic Zinc Ferrite Nanoparticles by Modified Ultrasonic Assisted Co-precipitation Method. ECS J. Solid State Sci. Technol..

[46] Lund A., Sagstuen E., Sanderud A., Maruani J. (2009). Relaxation-Time Determination from Continuous-Microwave Saturation of EPR Spectra. Radiat. Res..

[47] Li M., Wang X. (2025). Beating the Size-Dependent Limit with Spin–Lattice Coupling in Nanomagnetism. J. Am. Chem. Soc..

[48] Fittipaldi M., Innocenti C., Ceci P., Sangregorio C., Castelli L., Sorace L., Gatteschi D. (2011). Looking for quantum effects in magnetic nanoparticles using the molecular nanomagnet approach. Phys. Rev. B.

[49] Misra S.K., Andronenko S.I., Thurber A., Punnoose A., Nalepa A. (2014). An X- and Q-band Fe^3+^ EPR study of nanoparticles of magnetic semiconductor Zn_1−x_Fe_x_O. J. Magn. Magn. Mater..

[50] Radu L., Caruntu D., White M., Wiley J., Connor C.J.O., Hanson P. (2006). Ligand-dependent changes in the SPR of magnetic nanoparticles. 2006 NSTI Nanotechnology Conference and Trade Show—NSTI Nanotech 2006 Technical Proceedings.

[51] Piekarz P., Parlinski K., Oleś A.M. (2007). Origin of the Verwey transition in magnetite: Group theory, electronic structure, and lattice dynamics study. Phys. Rev. B.

[52] Stankowski J., Kempiński W., Łoś S., Bednarski W., Waplak S., Micnas R. (2006). Two paramagnetic iron states at the Verwey phase transition in magnetite. J. Magn. Magn. Mater..

[53] Adler P., Jeglič P., Reehuis M., Geiß M., Merz P., Knaflič T., Komelj M., Hoser A., Sans A., Janek J. (2018). Verwey-type charge ordering transition in an open-shell p-electron compound. Sci. Adv..

[54] Ahmad Y., Raina B., Thakur S., Bamzai K.K. (2022). Magnesium and yttrium doped superparamagnetic manganese ferrite nanoparticles for magnetic and microwave applications. J. Magn. Magn. Mater..

[55] Monisha P., Priyadharshini P., Gomathi S.S., Pushpanathan K. (2021). Influence of Mn dopant on the crystallite size, optical and magnetic behaviour of CoFe_2_O_4_ magnetic nanoparticles. J. Phys. Chem. Solids.

[56] Victory M., Pant R.P., Phanjoubam S. (2020). Synthesis and characterization of oleic acid coated Fe–Mn ferrite based ferrofluid. Mater. Chem. Phys..

[57] Masur S., Zingsem B., Marzi T., Meckenstock R., Farle M. (2016). Characterization of the oleic acid/iron oxide nanoparticle interface by magnetic resonance. J. Magn. Magn. Mater..

[58] Koper A., Krzyminiewski R. (1985). Analysis of resonance excitations by linear transformation technique theory. Acta Magn..

[59] Krzyminiewski R., Kowalczyk R.M., Bielewicz-Mordalska A., Pająk Z., Czarnecki P. (1998). Computer enhancement of CW-EPR experimental spectra resolution as a new method in investigation of molecular dynamics in pyridinium tetrafluoroborate. J. Mol. Struct..

[60] Dobosz B., Krzyminiewski R., Koralewski M., Hałupka-Bryl M. (2016). Computer enhancement of ESR spectra of magnetite nanoparticles. J. Magn. Magn. Mater..

[61] Dobosz B., Krzyminiewski R., Kurczewska J., Schroeder G. (2017). The influence of surface modification, coating agents and pH value of aqueous solutions on physical properties of magnetite nanoparticles investigated by ESR method. J. Magn. Magn. Mater..

[62] Noginova N., Chen F., Weaver T., Giannelis E.P., Bourlinos A.B., Atsarkin V.A. (2007). Magnetic resonance in nanoparticles: Between ferro- and paramagnetism. J. Phys. Condens. Matter.

[63] Upadhyay R.V., Srinivas D., Mehta R.V. (2000). Magnetic resonance in nanoscopic particles of a ferrofluid. J. Magn. Magn. Mater..

[64] Thamarai Selvi E., Meenakshi Sundar S. (2017). Effect of size on structural, optical and magnetic properties of SnO_2_ nanoparticles. Mater. Res. Express.

[65] Erdem E. (2014). Microwave power, temperature, atmospheric and light dependence of intrinsic defects in ZnO nanoparticles: A study of electron paramagnetic resonance (EPR) spectroscopy. J. Alloys Compd..

[66] Galyametdinov Y.G., Sagdeev D.O., Voronkova V.K., Sukhanov A.A., Shamilov R.R. (2018). The dependence of paramagnetic and optical characteristics of Mn:CdS nanoparticles on high-temperature synthesis conditions. Mater. Res. Express.

[67] Naik R., Prashantha S.C., Nagabhushana H., Girish K.M. (2018). Electrochemical, photoluminescence and EPR studies of Fe^3+^ doped nano Forsterite: Effect of doping on tetra and octahedral sites. J. Lumin..

[68] Al Boukhari J., Zeidan L., Khalaf A., Awad R. (2019). Synthesis, characterization, optical and magnetic properties of pure and Mn, Fe and Zn doped NiO nanoparticles. Chem. Phys..

[69] Atkins T.M., Walton J.H., Singh M.P., Ganguly S., Janka O., Louie A.Y., Kauzlarich S.M. (2017). EPR and Structural Characterization of Water-Soluble Mn^2+^-Doped Si Nanoparticles. J. Phys. Chem. C.

[70] Daniel J.E., Weaver S.I., Matthias B.R., Golden R., George G.M., Kerpal C., Donley C.L., Jarocha L.E., Anderson M.E. (2024). Investigating Cu-Site Doped Cu–Sb–S Nanoparticles Using Photoelectron and Electron Paramagnetic Resonance Spectroscopy. J. Phys. Chem. C.

[71] Misra S.K., Andronenko S.I., Srinivasa Rao S., Chess J., Punnoose A. (2015). An X-band Co^2+^ EPR study of Zn_1−x_Co_x_O (x = 0.005–0.1) nanoparticles prepared by chemical hydrolysis methods using diethylene glycol and denaturated alcohol at 5K. J. Magn. Magn. Mater..

[72] Choudhury B., Dey M., Choudhury A. (2013). Defect generation, d-d transition, and band gap reduction in Cu-doped TiO_2_ nanoparticles. Int. Nano Lett..

[73] Smirnov A.I. (2008). Post-processing of EPR spectra by convolution filtering: Calculation of a harmonics’ series and automatic separation of fast-motion components from spin-label EPR spectra. J. Magn. Reson..

[74] Travin S.O., Kokorin A.I. (2022). Kinetic Analysis and Resolution of Overlapping EPR Spectra. Appl. Magn. Reson..

[75] Kara G., Malekghasemi S., Ozpolat B., Denkbas E.B. (2021). Development of novel poly-l-lysine-modified sericin-coated superparamagnetic iron oxide nanoparticles as siRNA carrier. Colloids Surf. A Physicochem. Eng. Asp..

[76] Jamir M., Islam R., Pandey L.M., Borah J.P. (2021). Effect of surface functionalization on the heating efficiency of magnetite nanoclusters for hyperthermia application. J. Alloys Compd..

[77] Kavas H., Kasapoğlu N., Baykal A., Köseoğlu Y. (2009). Characterization of NiFe_2_O_4_ nanoparticles synthesized by various methods. Chem. Pap..

[78] Nedelcu G.G., Nastro A., Filippelli L., Cazacu M., Iacob M., Rossi C.O., Popa A., Toloman D., Dobromir M., Iacomi F. (2015). Structural characterization of copolymer embedded magnetic nanoparticles. Appl. Surf. Sci..

[79] Adams S., Bonabi S., Allen A.L., Roseman G., Ramirez A.P., Millhauser G., Zhang J.Z. (2018). The effect of polymer and gold functionalization on the magnetic properties of magnetite nanoparticles. Biomed. Spectrosc. Imaging.

[80] Deliormanlı A.M., Almisned G., Tekin H.O. (2024). Nanoarchitectonics and properties of sol-gel-derived bioactive glasses containing maghemite@ZnO core-shell nanoparticles. Appl. Phys. A.

[81] Çimen D., Bereli N., Denizli A. (2020). Metal-chelated magnetic nanoparticles for protein C purification. Sep. Sci. Technol..

[82] Orhan H., Aktaş Uygun D. (2020). Immobilization of L-Asparaginase on Magnetic Nanoparticles for Cancer Treatment. Appl. Biochem. Biotechnol..

[83] Vasić K., Knez Ž., Konstantinova E.A., Kokorin A.I., Gyergyek S., Leitgeb M. (2020). Structural and magnetic characteristics of carboxymethyl dextran coated magnetic nanoparticles: From characterization to immobilization application. React. Funct. Polym..

[84] Lloveras V., Badetti E., Chechik V., Vidal-Gancedo J. (2014). Magnetic Interactions in Spin-Labeled Au Nanoparticles. J. Phys. Chem. C.

[85] Zhang M., Li X.H., Gong Y.D., Zhao N.M., Zhang X.F. (2002). Properties and biocompatibility of chitosan films modified by blending with PEG. Biomaterials.

[86] Hirota Y., Haida M., Mohtarami F., Takeda K., Iwamoto T., Shioya S., Tsuji C., Hasumi K., Nakazawa H. (2000). Implication of ESR signals from ceruloplasmin (Cu^2+^) and transferrin (Fe^3+^) in pleural effusion of lung diseases. Pathophysiology.

[87] Preoteasa E.A., Schianchi G., Camillo Giori D., Duliu O.G., Butturini A., Izzi G. (2013). Unexpected Detection of Low- and High-Spin Ferrihemoglobin Derivatives in Blood Serum of Polytransfused Patients with Homozygous β-Thalassemia under Chelation Therapy. An EPR Study. Dig. J. Nanomater. Biostruct..

[88] Shukla A.K. (2019). Electron Spin Resonance Spectroscopy in Medicine.

[89] Kubiak T., Krzyminiewski R., Dobosz B., Schroeder G., Kurczewska J., Hałupka-Bryl M. (2015). A study of magnetite nanoparticles in whole human blood by means of electron paramagnetic resonance. Acta Bio-Opt. Inform. Medica Inżynieria Biomed..

[90] Gamarra L.F., Pontuschka W.M., Amaro E., Costa-Filho A.J., Brito G.E.S., Vieira E.D., Carneiro S.M., Escriba D.M., Falleiros A.M.F., Salvador V.L. (2008). Kinetics of elimination and distribution in blood and liver of biocompatible ferrofluids based on Fe_3_O_4_ nanoparticles: An EPR and XRF study. Mater. Sci. Eng. C.

[91] Kaczmarek K., Hornowski T., Dobosz B., Józefczak A. (2018). Influence of Magnetic Nanoparticles on the Focused Ultrasound Hyperthermia. Materials.

[92] Kubiak T. (2024). The Influence of Blood and Serum Microenvironment on Spin-Labeled Magnetic Nanoparticles. Magnetism.

[93] Chechik V., Wellsted H.J., Korte A., Gilbert B.C., Caldararu H., Ionita P., Caragheorgheopol A. (2004). Spin-labelled Au nanoparticles. Faraday Discuss..

[94] Stoll S., Schweiger A. (2006). EasySpin, a comprehensive software package for spectral simulation and analysis in EPR. J. Magn. Reson..

[95] Stoll S., Schweiger A. (2007). EasySpin: Simulating cw ESR spectra. Biol. Magn. Reson..

[96] Dobosz B., Krzyminiewski R., Kurczewska J., Schroeder G. (2017). The dynamics of functionalized magnetite nanoparticles in various solutions studied by ESR method. Mater. Chem. Phys..

[97] Krzyminiewski R., Dobosz B., Krist B., Schroeder G., Kurczewska J., Bluyssen H.A.R. (2020). ESR Method in Monitoring of Nanoparticle Endocytosis in Cancer Cells. Int. J. Mol. Sci..

[98] Saeidpour S., Lohan S.B., Anske M., Unbehauen M., Fleige E., Haag R., Meinke M.C., Bittl R., Teutloff C. (2017). Localization of dexamethasone within dendritic core-multishell (CMS) nanoparticles and skin penetration properties studied by multi-frequency electron paramagnetic resonance (EPR) spectroscopy. Eur. J. Pharm. Biopharm..

[99] Haag S.F., Chen M., Peters D., Keck C.M., Taskoparan B., Fahr A., Teutloff C., Bittl R., Lademann J., Schäfer-Korting M. (2011). Nanostructured lipid carriers as nitroxide depot system measured by electron paramagnetic resonance spectroscopy. Int. J. Pharm..

[100] Cheng T.-M., Li R., Kao Y.-C.J., Hsu C.-H., Chu H.-L., Lu K.-Y., Changou C.A., Chang C.-C., Chang L.-H., Tsai M.-L. (2020). Synthesis and characterization of Gd-DTPA/fucoidan/peptide complex nanoparticle and in vitro magnetic resonance imaging of inflamed endothelial cells. Mater. Sci. Eng. C.

[101] Hałupka-Bryl M., Bednarowicz M., Dobosz B., Krzyminiewski R., Zalewski T., Wereszczyńska B., Nowaczyk G., Jarek M., Nagasaki Y. (2015). Doxorubicin loaded PEG-b-poly(4-vinylbenzylphosphonate) coated magnetic iron oxide nanoparticles for targeted drug delivery. J. Magn. Magn. Mater..

[102] Davies M.J. (2016). Detection and characterisation of radicals using electron paramagnetic resonance (EPR) spin trapping and related methods. Methods.

[103] Jeong M.S., Yu K.-N., Chung H.H., Park S.J., Lee A.Y., Song M.R., Cho M.-H., Kim J.S. (2016). Methodological considerations of electron spin resonance spin trapping techniques for measuring reactive oxygen species generated from metal oxide nanomaterials. Sci. Rep..

[104] Dai Z., Tang J., Gu Z., Wang Y., Yang Y., Yang Y., Yu C. (2020). Eliciting Immunogenic Cell Death via a Unitized Nanoinducer. Nano Lett..

[105] Zhang H., Li S., Liu Y., Yu Y., Lin S., Wang Q., Miao L., Wei H., Sun W. (2020). Fe_3_O_4_@GO magnetic nanocomposites protect mesenchymal stem cells and promote osteogenic differentiation of rat bone marrow mesenchymal stem cells. Biomater. Sci..

[106] Liu Y., Quan X., Li J., Huo J., Li X., Zhao Z., Li S., Wan J., Li J., Liu S. (2023). Liposomes embedded with PEGylated iron oxide nanoparticles enable ferroptosis and combination therapy in cancer. Natl. Sci. Rev..

[107] Zhang C., Ren J., He J., Ding Y., Huo D., Hu Y. (2018). Long-term monitoring of tumor-related autophagy in vivo by Fe_3_O_4_NO· nanoparticles. Biomaterials.

[108] Silva M.A.S., Romo A.I.B., Abreu D.S., Carepo M.S.P., Lemus L., Jafelicci M., Paulo T.F., Nascimento O.R., Vargas E., Denardin J.C. (2018). Magnetic nanoparticles as a support for a copper (II) complex with nuclease activity. J. Inorg. Biochem..

[109] Moreno Maldonado A.C., Winkler E.L., Raineri M., Toro Córdova A., Rodríguez L.M., Troiani H.E., Mojica Pisciotti M.L., Mansilla M.V., Tobia D., Nadal M.S. (2019). Free-Radical Formation by the Peroxidase-Like Catalytic Activity of MFe_2_O_4_ (M = Fe, Ni, and Mn) Nanoparticles. J. Phys. Chem. C.

[110] Chen Z., Yin J.-J., Zhou Y.-T., Zhang Y., Song L., Song M., Hu S., Gu N. (2012). Dual Enzyme-like Activities of Iron Oxide Nanoparticles and Their Implication for Diminishing Cytotoxicity. ACS Nano.

[111] Valgimigli L., Baschieri A., Amorati R. (2018). Antioxidant activity of nanomaterials. J. Mater. Chem. B.

[112] Zhang Z., He X., Zhou C., Reaume M., Wu M., Liu B., Lee B.P. (2020). Iron Magnetic Nanoparticle-Induced ROS Generation from Catechol-Containing Microgel for Environmental and Biomedical Applications. ACS Appl. Mater. Interfaces.

[113] Lazarova D., Semkova S., Zlateva G., Tatsuya H., Aoki I., Bakalova R. (2021). Quantum Sensors To Track Total Redox-Status and Oxidative Stress in Cells and Tissues Using Electron-Paramagnetic Resonance, Magnetic Resonance Imaging, and Optical Imaging. Anal. Chem..

[114] Elas M., Krzykawska-Serda M., Gonet M., Kozińska A., Płonka P.M., Shukla A.K. (2019). Electron Paramagnetic Resonance Imaging-Solo and Orchestra. Medical Imaging Methods: Recent Trends.

[115] Danhier P., De Preter G., Magat J., Godechal Q., Porporato P.E., Jordan B.F., Feron O., Sonveaux P., Gallez B. (2014). Multimodal cell tracking of a spontaneous metastasis model: Comparison between MRI, electron paramagnetic resonance and bioluminescence. Contrast Media Mol. Imaging.

[116] Rana S., Chawla R., Kumar R., Singh S., Zheleva A., Dimitrova Y., Gadjeva V., Arora R., Sultana S., Sharma R.K. (2010). Electron paramagnetic resonance spectroscopy in radiation research: Current status and perspectives. J. Pharm. Bioallied Sci..

[117] Dobosz B., Krzyminiewski R., Schroeder G., Kurczewska J. (2016). Diffusion of functionalized magnetite nanoparticles forced by a magnetic field studied by EPR method. Curr. Appl. Phys..

[118] Bakker M.G., Fowler B., Bowman M.K., Patience G.S. (2020). Experimental methods in chemical engineering: Electron paramagnetic resonance spectroscopy-EPR/ESR. Can. J. Chem. Eng..

[119] Irfan M., Dogan N., Bingolbali A., Aliew F. (2021). Synthesis and characterization of NiFe2O4 magnetic nanoparticles with different coating materials for magnetic particle imaging (MPI). J. Magn. Magn. Mater..

[120] Eaton G.R., Eaton S., Barr D., Weber R.T. (2010). Quantitative EPR.

[121] Marnautov N.A., Serezhenkov V.A., Komissarova L.K., Tkachev N.A., Tatikolov A.S., Goloshchapov A.N., Vanin A.F. (2020). Evaluation of the Biodistribution of Magnetoliposomes in a Tumor and Organs of Mice by Electron Paramagnetic Resonance Spectroscopy. Biophysics.

[122] Chertok B., Cole A.J., David A.E., Yang V.C. (2010). Comparison of Electron Spin Resonance Spectroscopy and Inductively-Coupled Plasma Optical Emission Spectroscopy for Biodistribution Analysis of Iron-Oxide Nanoparticles. Mol. Pharm..

[123] Paulini F., Marangon A.R.M., Azevedo C.L., Brito J.L.M., Lemos M.S., Sousa M.H., Veiga-Souza F.H., Souza P.E.N., Lucci C.M., Azevedo R.B. (2022). In Vivo Evaluation of DMSA-Coated Magnetic Nanoparticle Toxicity and Biodistribution in Rats: A Long-Term Follow-Up. Nanomaterials.

[124] Partain B.D., Unni M., Rinaldi C., Allen K.D. (2020). The clearance and biodistribution of magnetic composite nanoparticles in healthy and osteoarthritic rat knees. J. Control. Release.

[125] Shimizu M., Yoshitomi T., Nagasaki Y. (2014). The behavior of ROS-scavenging nanoparticles in blood. J. Clin. Biochem. Nutr..

[126] Fuchs J., Groth N., Herrling T. (1998). Cutaneous Tolerance to Nitroxide Free Radicals in Human Skin. Free Radical Biol. Med..

[127] Lohan S.B., Saeidpour S., Colombo M., Staufenbiel S., Unbehauen M., Wolde-Kidan A., Netz R.R., Bodmeier R., Haag R., Teutloff C. (2020). Nanocrystals for Improved Drug Delivery of Dexamethasone in Skin Investigated by EPR Spectroscopy. Pharmaceutics.

[128] Huang W.-C., Tang I.C., Yang S.-T. (2007). Bacterial and Yeast Cultures—Process Characteristics, Products, and Applications. Bioprocessing for Value-Added Products from Renewable Resources.

[129] Krzyminiewski R., Dobosz B., Schroeder G., Kurczewska J. (2019). ESR as a monitoring method of the interactions between TEMPO-functionalized magnetic nanoparticles and yeast cells. Sci. Rep..

[130] Dobosz B., Gunia E., Kotarska K., Schroeder G., Kurczewska J. (2024). The Effect of a Magnetic Field on the Transport of Functionalized Magnetite Nanoparticles into Yeast Cells. Appl. Sci..

[131] Khmelinskii I., Makarov V.I. (2018). EPR hyperthermia of *S. cerevisiae* using superparamagnetic Fe_3_O_4_ nanoparticles. J. Therm. Biol..

